# Altered Channel Conductance States and Gating of GABA_A_ Receptors by a Pore Mutation Linked to Dravet Syndrome

**DOI:** 10.1523/ENEURO.0251-16.2017

**Published:** 2017-02-10

**Authors:** Ciria C. Hernandez, Weijing Kong, Ningning Hu, Yujia Zhang, Wangzhen Shen, Laurel Jackson, Xiaoyan Liu, Yuwu Jiang, Robert L. Macdonald

**Affiliations:** 1 Department of Neurology, Vanderbilt University Medical Center, Nashville, TN 37240-7915; 2 Department of Pediatrics, Peking University First Hospital, Beijing, China 100034

**Keywords:** conductance states, Dravet syndrome, *GABRG2*, gating, pore mutation, structural modeling

## Abstract

We identified a *de novo* missense mutation, P302L, in the γ-aminobutyric acid type A (GABA_A_) receptor γ2 subunit gene *GABRG2* in a patient with Dravet syndrome using targeted next-generation sequencing. The mutation was in the cytoplasmic portion of the transmembrane segment M2 of the γ2 subunit that faces the pore lumen. GABA_A_ receptor α1 and β3 subunits were coexpressed with wild-type (wt) γ2L or mutant γ2L(P302L) subunits in HEK 293T cells and cultured mouse cortical neurons. We measured currents using whole-cell and single-channel patch clamp techniques, surface and total expression levels using surface biotinylation and Western blotting, and potential structural perturbations in mutant GABA_A_ receptors using structural modeling. The γ2(P302L) subunit mutation produced an ∼90% reduction of whole-cell current by increasing macroscopic desensitization and reducing GABA potency, which resulted in a profound reduction of GABA_A_ receptor-mediated miniature IPSCs (mIPSCs). The conductance of the receptor channel was reduced to 24% of control conductance by shifting the relative contribution of the conductance states from high- to low-conductance levels with only slight changes in receptor surface expression. Structural modeling of the GABA_A_ receptor in the closed, open, and desensitized states showed that the mutation was positioned to slow activation, enhance desensitization, and shift channels to a low-conductance state by reshaping the hour-glass-like pore cavity during transitions between closed, open, and desensitized states. Our study revealed a novel γ2 subunit missense mutation (P302L) that has a novel pathogenic mechanism to cause defects in the conductance and gating of GABA_A_ receptors, which results in hyperexcitability and contributes to the pathogenesis of the genetic epilepsy Dravet syndrome.

## Significance Statement

Dravet syndrome is a catastrophic epileptic encephalopathy characterized by multiple types of seizures, impaired intellectual development, and cognitive decline. In general, only truncation mutations of γ-aminobutyric acid type A (GABA_A_) receptors have been reported in Dravet syndrome. We identified a single *de novo* mutation, P302L, that faces the pore lumen in the γ2 subunit, the most abundant GABA_A_ receptor subunit in the brain. GABA_A_ receptors mediate the majority of fast inhibitory neurotransmission and control network excitability in the brain. The present study showed that the γ2(P302L) subunit mutation caused ∼90% loss of function by altering the conduction pathway of the receptor during gating transitions among closed, open, and desensitized states, which ultimately enhances neuronal excitability.

## Introduction

Dravet syndrome (also known as severe myoclonic epilepsy in infancy, OMIM: 607208) is an epileptic encephalopathy of childhood that is characterized by multiple types of seizures that are often prolonged and particularly fever sensitive. With onset in the first year of life, *de novo* heterozygous missense mutations in *SCN1A*, which encodes the pore-forming α1 subunit of the sodium channel, are found in about 95% of patients with Dravet syndrome (Vadlamudi et al., 2010). Heterozygous nonsense mutations in the γ-aminobutyric acid type A (GABA_A_) receptor γ2 subunit gene, *GABRG2*, and missense mutations in the GABA_A_ receptor α1 subunit gene, *GABRA1*, are associated also with Dravet syndrome (Harkin et al., 2002; Hirose, 2006; Carvill et al., 2014). The *GABRG2* mutation, γ2(Q40X), caused truncation of the predicted mature *γ*2 subunit in the first amino acid, resulting in translation of no mature subunits and to activation of nonsense-mediated mRNA decay (NMD) that reduced truncated signal peptide production (Huang et al., 2012; Ishii et al., 2014). In contrast, a nonsense mutation in the last exon of *GABRG2*, γ2(Q390X), which was located in the intracellular loop of the *γ*2 subunit between transmembrane segments M3 and M4, produced mRNA that was stable and not degraded by nonsense-mediated mRNA decay, and truncated subunits that were degraded slowly and incompletely by endoplasmic reticulum-associated degradation (Kang et al., 2009). The heterozygous *Gabrg2^+/Q390X^* knock-in mouse had a severe epilepsy phenotype that included spontaneous generalized tonic-clonic seizures and sudden death after seizures and accumulation and aggregation of mutant γ2(Q390X) subunits that may contribute to the chronic progressively declining clinical course of the epileptic encephalopathy (Kang et al., 2015).

**Figure 1.**
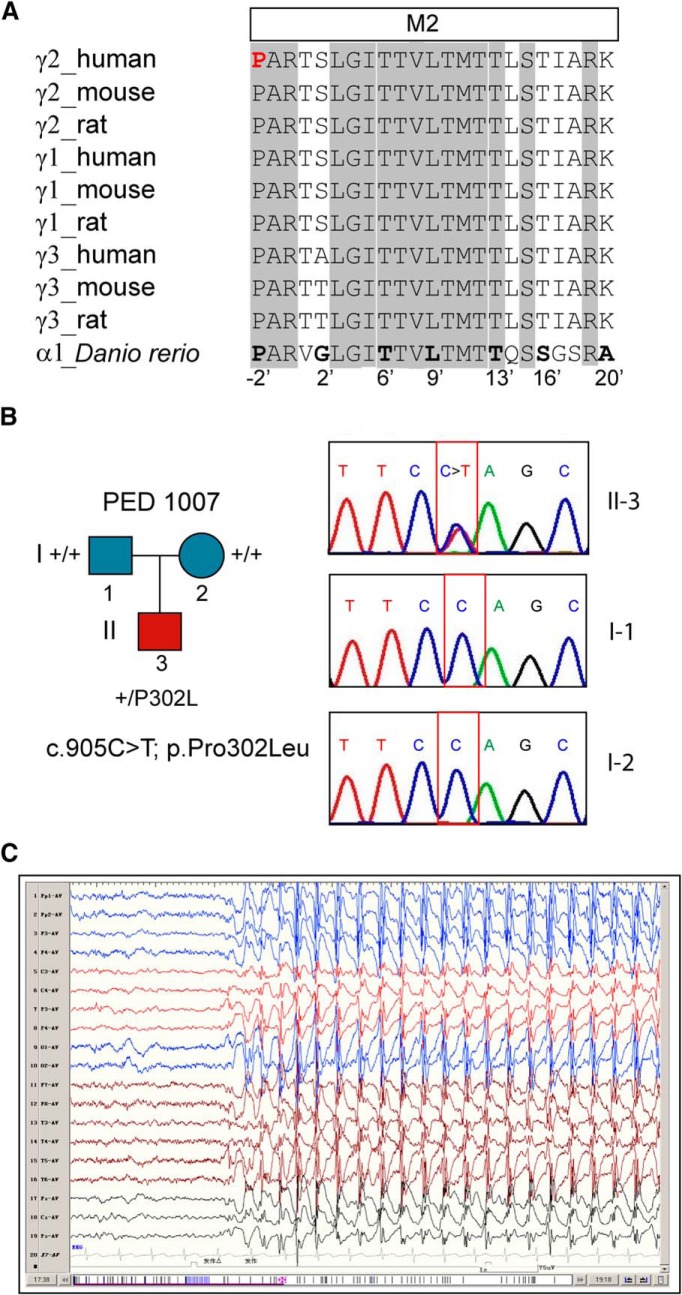
The *de novo* *GABRG2*(P302L) missense mutation was identified in a patient with Dravet syndrome. ***A***, Sequence alignments of the transmembrane segment M2 of GABA_A_ receptor γ1-3 subunits with the *D. rerio* GlyR receptor α1 subunit, highlighting the evolutionary conservation of the proline (shown in red) at the -2’ position. The residues in gray were conserved across all of the subunits, and pore-lining residues were numbered according to protein sequence and position in M2. ***B***, Pedigree and corresponding Sanger chromatograms of patient 1007 (II-3) and their unaffected parents (father as I-1 and mother as I-2) displayed the position of the substitution. The *de novo* substitution c.905C>T; p.Pro302Leu identified in this study was indicated in panel II-3 (red box) in comparison with panels I-1 and I-2 (blue boxes). ***C***, Representative EEG recorded during a seizure at 4 years of age showing generalized 2-3 Hz spike and poly-spike waves. PED, pedigree.

GABA_A_ receptor γ2 subunits, which belong to the Cys-loop ion channel superfamily, are formed by an ∼200 residue N-terminal extracellular domain and four transmembrane segments, M1 to M4, that are homologous to the glutamate-gated chloride channel (GluCL) (Hibbs and Gouaux, 2011), and the glycine receptor (GlyR) α1 subunit (Du et al., 2015). Pentameric assembly of GABA_A_ receptor α1, β3, and γ2 subunits form the most abundant GABA_A_ receptor subtype in the brain (Farrant and Nusser, 2005) that mediates the majority of fast inhibitory neurotransmission and controls network excitability in the brain. To date, only 8 missense mutations in the γ2 subunit are associated with mild forms of epilepsy syndromes and are located mainly in the receptor N-terminal extracellular domain and in the outermost region of the M2 transmembrane segment (Baulac et al., 2001; Wallace et al., 2001; Audenaert et al., 2006; Shi et al., 2010; Lachance-Touchette et al., 2011; Carvill et al., 2013; Reinthaler et al., 2015). Using targeted next-generation sequencing, we identified a novel *de novo GABRG2* missense mutation, P302L, in a patient with Dravet syndrome. Multiple sequence alignments among *GABRG* genes showed that this mutated proline was located in the cytoplasmic entrance of the transmembrane segment M2 that contributes to the formation of the pore region of the channel and was completely conserved in the three γ subunits in humans and other species (Fig. 1*A*). *In vitro* analysis showed that the γ2(P302L) subunit mutation caused slight changes of surface expression, but mainly disrupted GABA_A_ receptor gating by reduction of GABA-evoked single-channel current amplitudes, slowing activation, enhancing desensitization, and decreasing single-channel open probability, all of which caused reduction of GABA_A_ receptor-mediated mISPCs. We propose a structural mechanism that leads to dysfunction of this channel. Through modeling of the channel structure in the closed, open, and desensitized states that govern the gating of the receptor, we found that the mutation reshaped the pore of the GABA_A_ receptor by altering the features of both outer and inner vestibules of the hour-glass-like pore cavity, and thus altering the conduction pathway. The findings presented here shed light into the molecular mechanisms of the pathogenesis of Dravet syndrome.

## Materials and Methods

### Patient, targeted next-generation, and Sanger sequencing

Genomic DNA was extracted from peripheral leukocytes from the patient (case 1007) and parents for segregation analysis. Written informed consent was obtained from all individual subjects of this study. The study was approved by the Peking University First Hospital Medical Ethics Committee. Custom-designed panels capturing the coding exons of *GABRG2* were synthetized using the Agilent SureSelect Target Enrichment technique. Targeted next-generation sequencing was subsequently performed on an Illumina GAIIx platform (Illumina) using a paired-end sequencing of 110 bp to screen for mutations as described previously (Kong et al., 2015). We used Sanger sequencing to confirm the origin of the mutation as being *de novo*.

### cDNA constructs and expression of recombinant GABA_A_ receptors

cDNAs encoding human α1, β3, and γ2L GABA_A_ receptor subunit subtypes (NM000806, NM021912, and NM198904, respectively) were subcloned into the plasmid expression vector pcDNA3.1 (Thermo Fisher Scientific) using standard techniques. The γ2L(P302L) subunit mutation was generated by site-directed mutagenesis using the QuikChange Site-Directed Mutagenesis kit (Agilent Technologies) and verified by sequencing. Human embryonic kidney cells (HEK293T) were grown in 100-mm tissue culture dishes (Corning) with DMEM, supplemented with 10% fetal bovine serum at 37&cenveo_unknown_entity_timesnewroman_00B0;C in 5% CO_2_/95% air and passaged every 3-4 d. For surface biotinylation experiments, 4 × 10^5^ cells were plated onto 60-mm diameter culture dishes. Twenty-four hours after plating, cells were transfected using polyethyleneimine (PEI, MW 40,000; Polysciences). For mock or single subunit expression, empty pcDNA3.1 vector was added to make a final cDNA transfection amount to 1.8 μg. For electrophysiology experiments, cells were plated onto 12-mm cover glass chips at 4 × 10^4^ in 35-mm diameter culture dishes, transfected after 24 h with 0.3-μg cDNA of each α1, β3, and γ2L subunits and 0.05 µg of EGFP (to identify transfected cells) using X-tremeGENE9 DNA transfection Reagent (Roche Diagnostics; 1.5 μl/μg cDNA). Recordings were obtained 48 h after transfection.

### Surface biotinylation and Western blotting

Surface and total expression levels of wild-type (wt) α1, β3, γ2L, and γ2L(P302L) subunits were determined as described previously (Lo et al., 2010; Gurba et al., 2012; Huang et al., 2012). Primary antibodies against human α1 subunits (N-terminal, clone BD24, Millipore; 2.5 g/ml), human β3 subunits (N-terminal, monoclonal, β2/3-PE, clone 62-3G1, Millipore; 2.5 g/ml), and human γ2 subunits (clone S96-55, Novus Biologicals) were used to detect GABA_A_ receptor subunits. For Western blot experiments, Na^+^/K^+^-ATPase protein was used as a loading control (0.2 g/ml, clone 464.6, ab7671, Abcam), and anti-mouse IRdye conjugated secondary antibodies (Li-Cor) were used in all cases. Membranes were scanned using the Li-Cor Odyssey system, and integrated intensities of bands were determined using Odyssey software.

### Electrophysiology

Whole-cell recordings from lifted HEK293T cells and cell attached single-channel recordings were obtained as previously described (Hernandez et al., 2011; Janve et al., 2016). For whole-cell recordings the internal solution consisted of: 153 mM KCl, 10 mM HEPES, 5 mM EGTA, 2 mM Mg-ATP, and 1 mM MgCl_2_.6H_2_O; pH 7.3, ∼300 mOsm. The external solution was composed of: 142 mM NaCl, 8 mM KCl, 10 D(+)-glucose, 10 mM HEPES, 6 mM MgCl_2_.6H_2_O, and 1 mM CaCl_2_; pH 7.4, ∼326 mOsm. This combination of external (1Na-external) and internal solutions produced a chloride equilibrium reversal potential (V_rev_) of ∼0 mV, and cells were voltage clamped at -20 mV. For the ionic selectivity current-voltage (I/V) experiments, the 142 mM NaCl of the external recording medium was replaced with 71 mM NaCl (a 50% reduction of Na concentration), and 142 mM sucrose was added to maintain isoosmolar conditions. This external solution (0.5Na-external) produced a chloride V_rev_ of ∼13 mV. Liquid junction potentials were calculated using Clampex’s Junction Potential Calculator and corrected by the Pipette Offset circuitry of the amplifier. The I/V experiments were performed by holding the cell membrane potential (in mV) at: -80, -60, -40, -20, 0, +20, +40, +60, and 4 s GABA_A_ receptor currents evoked by 1 mM GABA were recorded at each membrane potential. External solutions and drugs were gravity fed to a four-barrel square glass pipette connected to a SF-77B Perfusion Fast-Step system (Warner Instruments). The solution exchange time across the open electrode tip was ∼200-400 μs, and the exchange around lifted cells (∼8-12 pF) occurred within 800 μs, which was sufficiently fast for these experiments (Bianchi and Macdonald, 2002) and guaranteed rapid solution exchanges and accurate measurement of the kinetic properties of the receptor currents. Although series resistance errors were not compensated in this study, we rule out the possibility of underestimating the “true” peak amplitude and desensitization kinetics by using low resistance electrodes (∼1 MΩ) and cells that showed current amplitudes ∼5 nA (∼8-12 pF) at −20 mV holding potential as previously reported (Bianchi and Macdonald, 2002). Single-channel currents were recorded in an external solution containing: 140 mM NaCl, 5 mM KCl, 1 mM MgCl_2_, 2 mM CaCl_2_, 10 mM glucose, and 10 mM HEPES; pH 7.4. During recording, 1 mM GABA was added to the internal solution that consisted of: 120 mM NaCl, 5 mM KCl, 10 mM MgCl_2_, 0.1 mM CaCl_2_, 10 mM glucose, and 10 mM HEPES; pH 7.4. The intramicropipette potential was +80 mV. αβ-GABA_A_ receptor currents were blocked by adding 100 µM zinc. Single-channel conductance in the presence of 1 mM GABA and 100 µM zinc was determined by holding the transmembrane potential at +80 mV. All experiments were performed at room temperature (22–23°C).

Whole-cell and single-channel currents were amplified and low-pass filtered at 2 kHz using an Axopatch 200B amplifier, digitized at 10 kHz (whole-cell recordings) or 20 kHz (single-channel recordings) using Digidata 1550 and saved using pCLAMP 10.4 (Axon Instruments). Data were analyzed offline using Clampfit 10.4 software (Axon Instruments, TAC 4.2 and TACFit 4.2; Bruxton).

Macroscopic activation and deactivation current times (τ) were measured by application of 1 mM GABA for 10 ms, while desensitization and peak current amplitude by application of 1 mM GABA for 4 s. Activation, desensitization and deactivation current time (τ) courses were fitted using the Levenberg–Marquardt least squares method with up to four component exponential functions of the form ∑*a_n_*exp(–*t*/τ*_n_*) + *C*, where *n* is the number of the exponential components, *t* is time, *a* is the relative amplitude, τ*_n_* is the time constant, and *C* is the residual current at the end of the GABA application. Additional components were accepted only if they significantly improved the fit, as determined by an *F* test on the sum of squared residuals. The time course of deactivation was summarized as a weighted time constant, defined by the following expression: ∑*a_n_*τ*_n_*/∑*a_n_*. The extent of desensitization was measured as (fitted peak current – fitted steady-state current)/(fitted peak current).

GABA peak current-voltage plots were fitted to a first order polynomial function, and V_rev_ values were read directly from the fitted current-voltage plots for each cell. Peak current responses obtained with 0.5Na solutions and recorded with holding potentials from -80 to -40 mV were excluded from the fitting analysis as they showed outward rectification. GABA_A_ receptor current concentration-response curves were fitted using GraphPad Prism version 6.07 for Windows (GraphPad Software). We used a nonlinear regression Hill equation of the form E = E_basal_ + (E_MAX_-E_basal_)/(1 + 10^((LogEC_50_-X)*Hill slope)), where E is the fractional response of the GABAR-gated currents, E_max_ is the maximal response, X is [GABA], EC_50_ is the [GABA] at which response = 50% of maximal response, and the Hill slope (*nH*). Thus, the *nH* for wt and γ2(P302L) GABA_A_ receptors were 1.17 ± 0.25 and 2.60 ± 0.88, respectively. Inhibition of 1 mM GABA_A_ receptor evoked currents by 10 µM zinc was measured by preapplication for 10 s followed by coapplication with GABA for 4 s. GABA and zinc were obtained from Sigma.

Single-channel open and closed events were analyzed using the 50% threshold detection method and visually inspected before accepting the events. Single-channel openings occurred as bursts of one or more openings or cluster of bursts. Bursts were defined as one or more consecutive openings that were separated by closed times that were shorter than a specified critical duration (***t***_crit_) prior to and following the openings (Twyman et al., 1990). A ***t***_crit_ duration of 5 ms was used in the current study. Clusters were defined as a series of bursts preceded and followed by closed intervals longer than a specific critical duration (***t***_cluster_). A ***t***_cluster_ of 10 ms was used in this study. Open and closed time histograms as well as amplitude histograms were generated using TACFit 4.2 (Bruxton). Single-channel amplitudes (***i***) were calculated by fitting all-point histograms with single- or multi-Gaussian curves. The difference between the fitted “closed” and “open” peaks was taken as ***i***. Duration histograms were fitted with exponential components in the form: ∑(***A_i_/***τ***_i_***)exp(–***t***/τ***_i_***), where ***A***and τ represent the relative area and time constant of the ***I***component, respectively, and ***t***is the time. The mean open time was then calculated as follows: ∑***A_i_***τ***_i_***. The number of components required to fit the duration histograms was increased until an additional component did not significantly improve the fit (Fisher and Macdonald, 1997). Single-channel current-voltage plots were fitted to a linear regression function and conductance values were extracted from the slopes of the best-fit line using GraphPad Prism version 6.07 for Windows (GraphPad Software).

### Primary cortical neuron culture

Mouse cortical neurons were obtained from embryonic day 17.5 mouse pups (four to eight) of either sex. C57BL/6 mice ages 2-4 months were used in schedule breeding 17 days prior to the day of neuron isolation (Huang et al., 2014). Two female mice were used after confirmation of pregnancy by detection of a vaginal plug. Briefly, dissociated cells were plated at a density of 6.5 × 10^4^ cells/cm2 onto 12-mm round coverslips in 24-well plates coated with poly-L-ornithine (0.5 mg/ml; Sigma). Cortical neurons were incubated at 37°C in 5% CO_2_ incubator and maintained in serum-free Neurobasal medium (Gibco) supplemented with B27 supplement (Gibco), glutamine (Gibco), and penicillin/streptomycin (Gibco, 20 U/ml). Cultured neurons were transfected at DIV12 with 2 µg of γ2L or γ2L(P302L) and 0.5 µg of EGFP using X-tremeGENE9 DNA transfection reagent. Recording were obtained at DIV 19-21. All animal procedures were performed in accordance with Vanderbilt University Medical Center animal care committee's regulations.

### GABA_A_ receptor-mediated miniature IPSCs (mIPSCs) and analysis

Whole-cell patch-clamp recordings were obtained at room temperature (22–23°C) at a holding potential of −60 mV. Neurons were perfused with an extracellular solution containing 145 mM NaCl, 3 mM KCl, 1.5 mM CaCl_2_,1 mM MgCl_2_.6H_2_O, 10 mM glucose, and 10 mM HEPES; pH 7.4, ∼320 mOsm. The internal solution consisted of 135 mM CsCl, 2 mM MgCl_2_.6H_2_O, 10 mM EGTA, 5 Mg-ATP, and 10 mM HEPES; pH 7.2, ∼300 mOsm. GABA_A_ receptor-mediated mIPSCs were recorded by blocking both excitatory neurotransmission and action potential generation. Thus, tetrodotoxin (0.5 μM), d(−)-2-amino-5-phosphonopentanoate (40 μM), and 6-cyano-7-nitroquinoxaline-2,3-dione (10 μM) were added the extracellular solution. Whole-cell currents were amplified and low-pass filtered at 2 kHz using an Axopatch 200B amplifier, digitized at 10 kHz using Digidata 1550, and saved using pCLAMP 10.4 (Axon Instruments). Data were analyzed offline using Clampfit 10.4 software (Axon Instruments).

Analysis of GABA_A_ receptor-mediated mIPSCs were obtained using the Clampfit 10.4 data analysis module. Detection of mIPSCs was determined from all automatically detected events in a given 200-s recording period. For kinetic analysis, the mIPSCs were automatically detected by the program initially and then manually analyzed based on the criteria that only single-event mIPSCs with a stable baseline, rising phase (10-90% rise time), and exponential decay were chosen during visual inspection of the recording trace. Double- and multiple-peak mIPSCs were excluded. Events of low amplitude (<15 pA) were discarded from this analysis. For each neuron, 1000 individual mIPSC events were recorded. Cumulative histograms of the peak currents were compared using a Kolmogorov–Smirnov (K-S) test with a significance value of *p* < 0.05.

### Structural modeling, simulation, and channel pore characterization

Three-dimensional structural models of the GABA_A_ receptor in the open, closed, and desensitized states were modelled using the electron cryo-microscopy of the *Danio rerio* GlyR subunit α1 structures (Du et al., 2015) in the open (3JAE), closed (3JAD), and desensitized (3JAF) conformations as templates. GABA_A_ receptor α1, β3, and γ2 subunit raw sequences in FASTA format were uploaded to the Swiss-PdbViewer 4.10 server (Schwede et al., 2003) for template searching against the ExPDB database (ExPASy, http://www.expasy.org/). The initial sequence alignments between GABA_A_ receptor α1, β3, and γ2 subunits and *D. rerio* GlyR α1 subunits in the open (3JAE), closed (3JAD), and desensitized (3JAF) states were generated with full-length multiple alignments using ClustalW. Sequence alignments were inspected manually to assure accuracy among structural domains solved from the template. Because the long M3/M4 cytoplasmic loop of the GABA_A_ receptor subunits was absent in the solved GlyR structures, the corresponding fourth transmembrane segments (M4) were misaligned onto the template. Consequently, the M3/M4 cytoplasmic loop was excluded from the modelling, and separate alignments were generated for the transmembrane segments M4. Then full-length multiple alignments were submitted for automated comparative protein modelling implemented in SWISS-MODEL (http://swissmodel.expasy.org/SWISS-MODEL.html). Before energy minimization, resulting 3D models of human GABA_A_ receptor α1, β3, and γ2 subunits in the three conformational states were inspected manually, their structural alignments confirmed, and evaluated for proper h-bonds, presence of clashes, and missing atoms using Molegro Molecular Viewer (CLC bio). Pentameric 3D GABA_A_ receptor models were generated by combining α1, β3, and γ2 structural models in the stoichiometry 2β:2α:1γ with the subunit arrangement β3-α1-β3-α1-γ2 in a counterclockwise order by superposition onto the *D. rerio* GlyR receptor in the open (3JAE), closed (3JAD), and desensitized (3JAF) conformation states. Neighborhood structural conformational changes within a radius of 6 Å of the mutated residue P302L in the γ2 subunit in the 3D structural models of the GABA_A_ receptor in the open, closed, and desensitized conformation states were modelled using Rosetta 3.1 (Smith and Kortemme, 2008) (https://kortemmelab.ucsf.edu). Up to 20 of the best-scoring structures were generated at each time by choosing parameters recommended by the application. Root mean squared (RMS) deviation was calculated between the initial (wt) structures and superimposed modelled (mutated) structures. For each 3D GABA_A_ receptor conformational state, the average RMS deviation over 10 low-energy structures was computed and conformational changes displayed among neighborhood structural domains. The detection and pore shape and visualization of the 3D structural models of the wt and mutated (P302L) GABA_A_ receptor in the open, closed, and desensitized conformation states were determined using PoreWalker (Pellegrini-Calace et al., 2009), a computational automated method available as a web-based resource (http://www.ebi.ac.uk/thornton-srv/software/PoreWalker/). From the PoreWalker outputs, a list of the identified pore-lining residues with their β-carbon coordinates along the pore axis (i.e., *x*-coordinates) and the given pore diameter profile of a channel were used to plot of pore diameters as a function of distance along the pore axis. In addition, we implemented ChExVis (Masood et al., 2015) (http://vgl.serc.iisc.ernet.in/chexvis/) to determine the pore radius profile and the list of the identified pore-lining residues of the transmembrane pore of the 3D structural models of the wt and the P302L in the open conformation state. The models were rendered using UCSF Chimera version 1.10 (Pettersen et al., 2004).

### Statistical analysis

Numerical data were expressed as mean ± SEM. Statistical analysis was performed using GraphPad Prism version 6.07 for Windows (GraphPad Software). Statistical significance was taken as *p* < 0.05, using unpaired two-tailed Student’s *t* test, one-way ANOVA with Dunnett’s and Tukey’s multiple comparisons test, or two-way ANOVA with Sidak’s multiple comparisons test as appropriate.

## Results

### A *de novo* mutation in *GABRG2* that encodes the GABA_A_ receptor γ2 subunit was associated with Dravet syndrome

Using a designed targeted sequencing panel, we identified a heterozygous *de novo* missense mutation in *GABRG2*, c.905C>T (encoding p.Pro302Leu), which affected the highly conserved transmembrane segment M2 that lines the pore domain of the GABA_A_ receptor γ2 subunit (Fig. 1*A*,*B*). The affected male (patient 1007) carrying the *de novo* missense γ2 subunit mutation c.905C>T; p.Pro302Leu (Fig. 1*B*) was diagnosed with Dravet syndrome. The phenotype included early psychomotor and language developmental delay and multiple seizure types (partial seizures, generalized tonic-clonic seizures, atypical absence seizures, and myoclonus) that were often prolonged. Neither parent had cognitive impairment or epilepsy. During his first year of life, he had seizures with febrile episodes, and his EEG was normal. However, he had frequent afebrile seizures after his first year. When he was 4 years old, the patient still had frequent atypical absence seizures with EEG showing bursts of bilateral 2- to 3-Hz spike waves and multispike and slow waves (Fig. 1*C*). The patient was treated with more than five antiepileptic drugs, which did not control the seizures. Therefore, his epilepsy syndrome was consistent with a diagnosis of Dravet syndrome.

### The GABA_A_ receptor γ2(P302L) subunit mutation substantially reduced GABA-evoked currents, enhanced their desensitization, and reduced GABA potency

**Figure 2.**
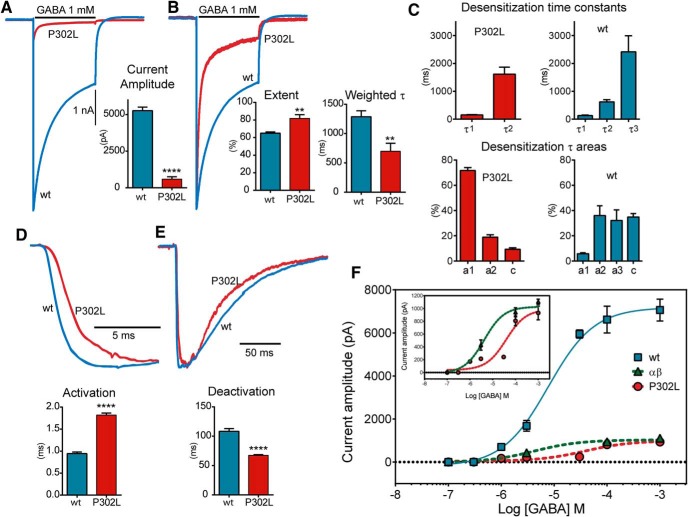
The *de novo* γ2(P302L) subunit mutation reduces GABA-activated currents and enhances desensitization. ***A***, ***B***, Representative GABA evoked-current traces obtained following rapid application of 1 mM GABA for 4 s to lifted HEK293T cells expressing wt γ2L and mutant γ2L(P302L) subunit-containing α1β3γ2L GABA_A_ receptors. Traces on the right (***B***) were normalized to illustrate the differences in desensitization between wt and mutant receptor currents. Bar graphs summarized the effects of wt and mutant GABA_A_ receptors on peak current amplitudes and extent and weighted τ of desensitization. ***C***, Bar graphs summarized the effects of wt and mutant GABA_A_ receptors on desensitization time constants (τ1, τ2, and τ3) and relative areas (a1, a2, and a3). ***D***, ***E***, Representative current traces showed activation (***D***) and deactivation (***E***) obtained following rapid application of 1 mM GABA for 10 ms to cells coexpressing α1 and β3 subunits with wt γ2L or mutant γ2L(P302L) subunits. The traces were normalized for clarity. Bar plots summarized the differences in activation and deactivation between wt and mutant receptors. ***F***, GABA_A_ receptor concentration-response curves for wt αβγ (solid blue line), αβ (dashed green lines), and mutant γ2L(P302L) subunits (dashed red lines) were obtained. The top left inside panel was a comparison between currents from receptors containing wt αβ and mutant αβγ2L(P302L) subunits. Values were expressed as mean ± SEM (*n* = 5-6 cells for each experimental condition). The data represented the summary of 17 cells with comparable capacitances (8-12 pF) recorded from three independent transfections. Values were expressed as mean ± SEM. Unpaired two-tailed Student’s *t* test relative to wt γ2L. ****p < 0.0001, **p < 0.01, respectively. See Table 1 for details.

To gain insight into the molecular mechanisms underlying the epilepsy syndrome, we determined how GABA_A_ receptor function was affected by the presence of the γ2(P302L) subunit mutation. Whole-cell currents were evoked from lifted HEK293T cells cotransfected with α1 and β3 subunits and wt γ2L or mutant γ2L(P302L) subunits by applying a saturating GABA concentration (1 mM) for 4 s and 10 ms using a rapid exchange system (Fig. 2). Peak GABA-evoked current amplitudes recorded from cells coexpressing α1β3γ2L(P302L) subunits were reduced to ∼10% of those from wt α1β3γ2L receptors (*p* < 0.0001; Fig. 2*A*, Table 1) .

**Table 1.** Effects of γ2(P302L) subunit mutation on α1β3γ2L receptor channel function

**Whole-cell currents**	***α1β3γ2L***	***α1β3γ2L(P302L)***	***p*^a^**	***αβ*^b^**
Current amplitude, pA	5264 ± 244 (*n* = 12)	590 ± 164 (*n* = 12)	<0.0001	1790 ± 232^e^ (*n* = 10)
Desensitization extent, %	65 ± 1 (*n* = 12)	82 ± 4 (*n* = 12)	0.0025	79 ± 5^g^ (*n* = 10)
Desensitization weight τ, ms	1289 ± 100 (*n* = 12)	698 ± 137 (*n* = 12)	0.0023	1057 ± 265^g^ (*n* = 9)
Zinc inhibition, %	12 ± 2 (*n* = 11)	82 ± 2 (*n* = 8)	<0.0001	95 ± 2^f^ (*n* = 5)
Activation τ, ms	0.94 ± 0.04 (*n* = 19)	1.82 ± 0.05 (*n* = 9)	<0.0001	3.30 ± 0.13^c^ (*n* = 14)
Deactivation τ, ms	108.5 ± 4.49 (*n* = 11)	67.31 ± 1.33 (*n* = 9)	<0.0001	177.6 ± 27.84^d^ (*n* = 12)
**Desensitization time constants**			***p*^h^**	***p*^i^**
τ_1_, ms	129.5 ± 17.9 (*n* = 5)	151.3 ± 11.7 (*n* = 5)	0.0020	
τ_2_, ms	620.3 ± 76.7 (*n* = 5)	1616 ± 252 (*n* = 5)	
τ_3_, ms	2417 ± 577 (*n* = 5)	-		0.2103
a_1_, %	6 ± 1 (*n* = 5)	72 ± 2 (*n* = 5)	<0.0001	
a_2_, %	36 ± 8 (*n* = 5)	19 ± 2 (*n* = 5)		
a_3_, %	32 ± 8 (*n* = 5)	-		
c, %	35 ± 3 (*n* = 5)	9 ± 1 (*n* = 5)	

Macroscopic parameters were obtained from whole-cell currents recorded from lifted cells, which were voltage-clamped at -20 mV and exposed with 1 mM GABA for 4 s. Desensitization extent and desensitization τ refer to the percentage of current amplitude at the end of GABA applications and weighted desensitization time constant, respectively. Activation and deactivation τ refer to weighted activation and deactivation time constant, respectively, when applying 1 mM GABA for 10 ms. Values reported are mean ± SEM. ^a^Unpaired two-tailed Student’s *t* test relative to α1β3γ2L. ^b^One-way ANOVA with Tukey’s multiple comparisons test was used for significance between αβ and wt α1β3γ2L or mutant α1β3γ2L(P302L) receptors. The differences relative to α1β3γ2L(P302L) were reported. ^c^*p* < 0.0001, ^d^*p* < 0.001, ^e^*p* < 0.01, ^f^*p* < 0.05, and ^g^*p* > 0.05, respectively. ^h^Two-way ANOVA with Sidak’s multiple comparisons test relative to τ1 and τ2 (both wt and mutant). ^i^Two-way ANOVA with Sidak’s multiple comparisons test relative to τ1 (both wt and mutant), τ2 (mutant), and τ3 (wt).

To further determine whether the γ2L(P302L) subunit mutation altered the gating of γ2L(P302L) subunit-containing GABA_A_ receptors, we examined the desensitization, activation, and deactivation rates of macroscopic whole-cell currents, properties that represent transitions among the closed, open, and desensitized states of the receptor. Currents from cells coexpressing α1β3γ2L(P302L) subunits with α1 and β3 subunits were strongly desensitized (∼80%; Fig. 2*B*,*C*), slowly activated (∼2 times slower than wt receptor currents; Fig. 2*D*), and rapidly deactivated (∼2 times faster than wt receptor currents; Fig. 2*E*, Table 1). Moreover, while wt currents desensitized with three exponential components (τ1, τ2, τ3) as reported before (Bianchi et al., 2007), γ2L(P302L) currents desensitized with only two exponential components (τ1 and τ2; Fig. 2*C*). The first desensitization time constant (τ1) was not different from wt, but its relative contribution increased from 6 to 72% (*p* < 0.0001; Table 1), which may account for the acceleration of the desensitization weighted τ. Interestingly, the second time constant (τ2) of the mutant receptor was not different from the longest wt time constant (τ3) (*p* < 0.2103; Table 1), which suggested the mutant lacks the second or intermediate time constant (τ2) (*p* < 0.0020; Table 1).

To determine whether the changes observed in receptor gating altered GABA_A_ receptor efficacy and/or potency, we measured the effects of mutant γ2L(P302L) subunits on α1β3γ2L GABA_A_ receptor concentration-response curves (Fig. 2*F*). Thus, macroscopic peak currents were evoked by applying various concentrations of GABA for 4 s to wt and mutant receptors. For wt receptors, the EC_50_ for current stimulation was 7.50 ± 0.77 µM, and the maximal current was 7038 ± 302 pA (*n* = 5-6). The γ2L(P302L) subunit mutation caused an ∼6-fold right shift of the EC_50_ (50.26 ± 0.82 µM), with a substantial reduction of peak current to ∼13% of the maximal response to GABA (931.2 ± 57.6 pA, *n* = 5-6). When comparing the maximal response to GABA between mutant ternary α1β3γ2L receptors (see above) and wt binary α1β3 receptors (1204 ± 235 pA, *n* = 5-6), their efficacy was similar, but binary α1β3 receptor potency (see above) was 10-fold higher than mutant ternary α1β3γ2L receptors (5.20 ± 0.47 µM), which was comparable with previous results (Wooltorton et al., 1997). These results suggest that, although the γ2L(P302L) subunit mutation conferred characteristics to ternary receptors that resembled those of binary receptors, their kinetic defects were distinguishable.

### The *de novo* γ2(P302L) subunit mutation slightly reduced surface levels of GABA_A_ receptor α1, β3, and γ2 subunits

**Figure 3.**
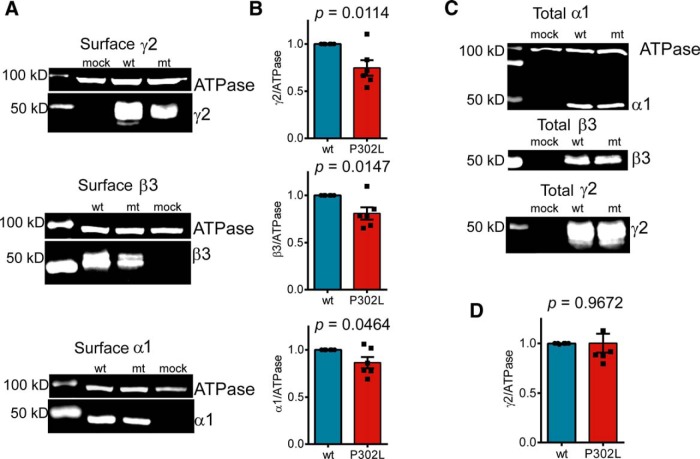
The γ2(P302L) subunit mutation causes slight changes in surface levels of GABA_A_ receptor subunits. ***A***, Wild-type (wt) γ2L or mutant (mt) γ2L(P302L) subunits were coexpressed with α1β3 subunits in HEK293T cells. Surface protein samples were biotinylated, isolated, separated by SDS-PAGE, and probed by anti-GABA_A_ receptor subunit and anti-ATPase antibodies. ***B***, Band intensities of the γ2, β3, and α1 subunits were normalized to the ATPase signal, and the summarized data were shown in the bar graphs. ***C***, Wild-type (wt) or mutant (mt) γ2L(P302L) subunits were cotransfected with α1β3 subunits into HEK293T cells, and total cell lysates were collected, analyzed by SDS-PAGE, and blotted by anti-α1, anti-β3, or anti-γ2 subunit and anti-ATPase antibodies. ***D***, Band intensity of γ2L subunits was normalized to the ATPase signal. Values were expressed as mean ± SEM. An unpaired *t* test was used to determine significance.

Changes in maximum GABA_A_ receptor currents caused by mutations may be due to deficits in expression of the subunits that assemble to form functional receptors on the cell surface. Therefore, we determined whether the γ2L(P302L) subunit mutation altered GABA_A_ receptor cell surface expression. We coexpressed α1, β3, and either wt γ2L or mutant γ2L(P302L) subunits in HEK293T cells and assessed surface and total expression levels of GABA_A_ receptor subunits. Relative to coexpressed γ2L, β3, and α1 subunits, surface levels of coexpressed γ2L(P302L), β3, and α1 subunits were slightly reduced to 0.75 ± 0.08 (*p* = 0.0114, *n* = 6), 0.81 ± 0.06 (*p* = 0.0147, *n* = 6), and 0.86 ± 0.06 (*p* = 0.0464, *n* = 6), respectively (Fig. 3*A*,*B*). Total cellular expression of α1 (0.87 ± 0.14, *n* = 3, *p* = 0.4058), β3 (0.92 ± 0.05, *n* = 3, *p* = 0.1572) (Fig. 3*C*), and γ2(P302L) (1.00 ± 0.09, *n* = 6, *p* = 0.9672) subunits was not altered (Fig. 3*C*,*D*).

Taken together, the γ2L(P302L) subunit mutation minimally disrupted α1, β3, and γ2 subunit surface expression, suggesting that the mutation minimally altered subunit oligomerization or receptor assembly. However, these data do not rule out the possibility that the mutation affected receptor subunit stoichiometry. To determine whether the receptor expressed in the membrane was a tertiary αβγ receptor or a combination of binary αβ and tertiary αβγ receptors, we compared the macroscopic kinetics of zinc-sensitive α1β3 receptors (Hosie et al., 2003) with relatively zinc-insensitive wt α1β3γ2L and mutant α1β3γ2L(P302L) receptors (Table 1). The fractional zinc inhibition of currents from cells coexpressing α1β3γ2L(P302L) subunits was different from that from cells coexpressing α1β3γ2L subunits (*p* < 0.0001) or from cells coexpressing α1β3 subunits (*p* < 0.05). Furthermore, currents from cells coexpressing α1β3γ2L(P302L) subunits activated and deactivated ∼2-3 times faster than α1β3 receptor currents (Table 1). Thus, α1β3γ2L(P302L) current kinetic properties were unique and different from those of wt α1β3γ2L or α1β3 receptors, which established that the γ2(P302L) subunit mutation substantially changed receptor gating, but not subunit stoichiometry. Although we cannot totally exclude the possibility of the presence of a small proportion of α1β3 receptors, these results suggested that the mutated γ2L(P302L) subunit was effectively assembled in the receptor and, once in the surface membrane, drastically reshaped the kinetic behavior of the receptor.

### Three-dimensional structural modeling of pentameric α1β3γ2 GABA_A_ receptors showed that the γ2(P302L) subunit mutation occurred where the desensitization-closed gate of Cys-loop receptors resides

**Figure 4.**
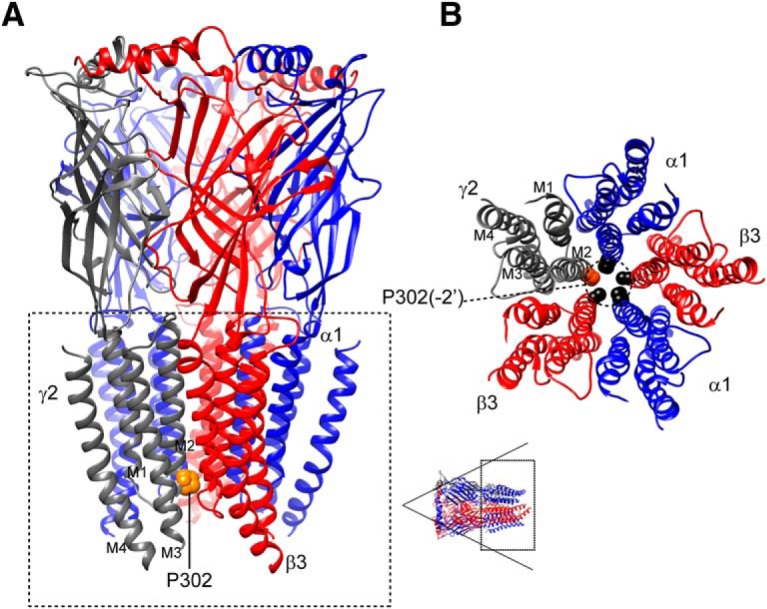
The *de novo* γ2(P302L) subunit mutation was an evolutionary conserved residue in the pore region of the GABA_A_ receptor. ***A***, A 3D structural model of the α1β3γ2 GABA_A_ receptor was displayed with the β subunits in red, α subunits in blue, and the γ subunit in gray. The γ2(P302L) subunit mutation was mapped onto the structure and represented in orange. The dashed box represented the transmembrane domain of the receptor and transmembrane segments M1 to M4 were labeled in the γ2 subunit. ***B***, The transmembrane domain of 3D structural model of the α1β3γ2 GABA_A_ receptor, with residues at positions -2′ (dashed circle) in each transmembrane segment M2 was displayed as spheres and colored by subunit (γ2 in orange, and α1 and β3 in black). Subunits α1 (blue), β3 (red), and γ2 (gray) were labeled, and transmembrane segments M1 to M4 were labeled in the γ2 subunit. A 3D model was viewed from the extracellular side as shown in the lower left corner (dashed box); for clarity, the N-terminal extracellular domain was not shown.

Three-dimensional structural modeling of the pentameric α1β3γ2 GABA_A_ receptor showed that the γ2(P302L) subunit mutation occurred at the -2’ position of the transmembrane segment M2 (Figs. 1*A* and 4*B* ), which was at the intracellular entrance of the pore (Fig. 4*A*). Recent x-ray structures of the GluCL (Hibbs and Gouaux, 2011) and the electron cryo-microscopy of the GlyR (Du et al., 2015) demonstrated that the desensitization-gate of Cys-loop receptors resides at the intracellular entrance of the pore (Gielen et al., 2015), where the -2’P302 mutation was located, which represented the second site of constriction within the pore. These findings suggest a molecular mechanism by which the γ2(P302L) subunit mutation mainly reduced GABA-evoked currents and induced fast desensitization of GABA_A_ receptors.

**Figure 5.**
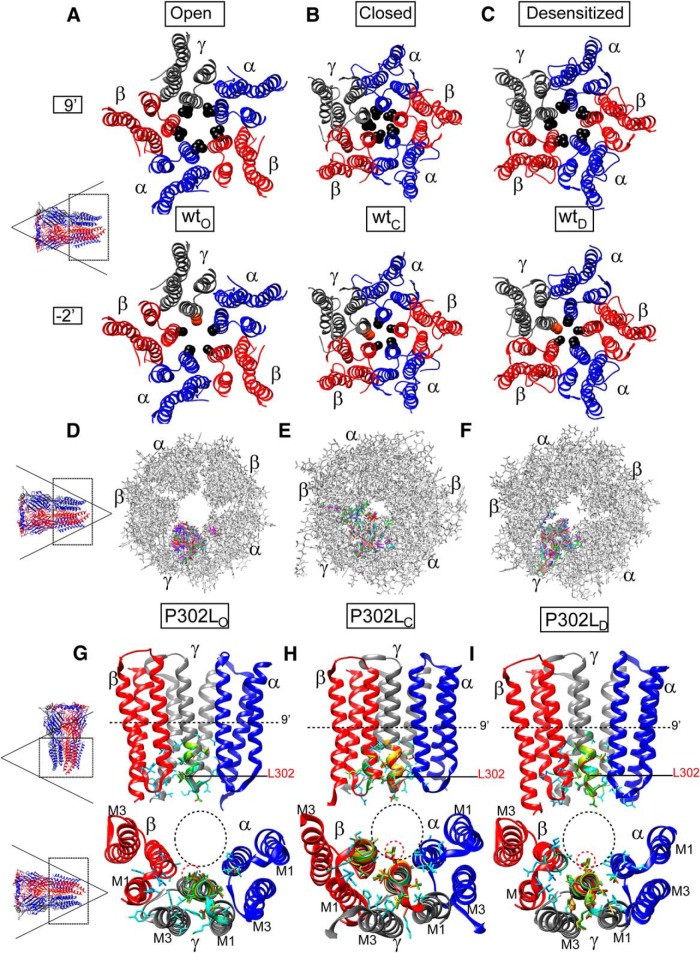
The pore mutation P302L perturbed the conduction pathway of GABA_A_ receptors. ***A-C***, Transmembrane domains (M1 to M4) of 3D structural models of the α1β3γ2 GABA_A_ receptor in the open (***A***), closed (***B***), and desensitized (***C***) conformational states were viewed from the extracellular side and displayed the β subunits in red, α subunits in blue, and γ subunit in gray. Side chains of the pore-lining residues at 9’(in black) and -2’ (P302 in orange, other residues in black) positions were shown within the ion channel pore of the receptor. ***D-F***, Superimposed 10 best-scoring 3D transmembrane domains of GABA_A_ receptors in the open (***D***), closed (***E***), and desensitized (***F***) states modelled between the initial wt and P302L mutated structures were viewed from the cytoplasmic side. The modelled structures were in stick representation. The wt GABA_A_ receptor structure was in gray. The structural rearrangements in side chain residues that differ among the wt and the mutated structures (RMS ≥ 0.5 Å) were represented as a different color. ***G-I***, Superimposed 10-best-scoring transmembrane domains of P302L structures in the open (***G***), closed (***H***), and desensitized (***I***) states were seen parallel to the membrane (top panels) and from the cytoplasmic side (bottom panels). Two subunits were removed for clarity. Perturbed neighborhood side chains within of 10 Å of L302 at the -2’ position were shown within the ion channel pore, and the structural perturbations that differ among the wt and mutated structures (RMS ≥ 0.5 Å) were represented in different colors. The wt-γ2 subunit structure was in gray. The channel gate at the 9’ position was represented as a dashed line in the top panels. The β and α subunits were in red and blue, respectively. In the bottom panels, dashed black circles represented the channel pore, and the location of the L302 residue was shown in red dashed circles. Lists of the residues perturbed by the L302 mutation were detailed in the text.

Considering that residues lining the transmembrane segment M2 of the receptor contribute to gating transitions between closed and open states, we propose that the mutant γ2(P302L) subunit might have different impacts on receptor structure in the closed, open, and desensitized states. Taking advantage of the electron cryo-microscopy of the *D. rerio* GlyR structures (Du et al., 2015) in the open (3JAE), closed (3JAD), and desensitized (3JAF) conformational states, we modelled wt α1β3γ2 and mutant α1β3γ2(P302L) receptors in the open (Fig. 5*A*,*D*), closed (Fig. 5*B*,*E*), and desensitized (Fig. 5*C*,*F*) states (see Materials and Methods for details). As described for the GlyR (Du et al., 2015), the α1β3γ2 GABA_A_ receptor pore exposed two sites of constriction, one at position 9’ (Fig. 5*A-C*, top panels), which corresponded to the activation/open gate, and the other in the cytoplasmic interface at position -2’ (Fig. 5*A-C*, lower panels), which represented the desensitization gate (Gielen et al., 2015). The α1β3γ2 GABA_A_ receptor modeling showed that these two gates were open or closed depending on the conformational state of the receptor. Structural modeling of α1β3γ2(P302L) receptors in the open (Fig. 5*D*), closed (Fig. 5*E*), and desensitized (Fig. 5*F*) states showed that the γ2(P302L) subunit mutation caused rearrangements of the side chain residues confined to the transmembrane segment M2 of the γ2 subunit and propagated to the α1 and β3 neighboring subunits. We computed that the rearrangements of the subunit’s secondary structure between the wt and mutant structural modeling had RMS deviation values ≥0.5 Å, which were shown in rainbow colors in Figure 5*D-F*. In addition, the γ2(P302L) subunit modeling showed a repositioning of the side chains of the pore-lining residues of neighboring β3 and α1 subunits in between -2’ and 9’ positions (Fig. 5*G-I*), where the closed-desensitized and open gates are located.

The rearrangements observed in the side chains of the pore-lining residues were dependent on the conformational state of the receptor. In addition, the residues were perturbed differently depending on their localization within the pentameric receptor when taking into account its counterclockwise arrangement as β3(chain A)-α1(chain B)-β3(chain C)-α1(chain D)-γ2(chain E) subunits (chains refer to the subunits in the pentameric structure). Thus, in the open state (Fig. 5*G*), the perturbations were located mainly below the 9’ position, perturbing pore-lining residues in the M2 helices of α1D-γ2E-β3A neighbor subunits (α1V279-V287, γ2A300-T310, β3A271-L278), and in the M3 helix of γ2(L350-V360). In contrast, in the closed and desensitized states, all five β3A-α1B-β3C-α1D-γ2E subunits were altered (β3A and β3C, I267-T281; α1B and α1D, S278-F285; γ2E, S293-T310), compromising a greater number of residues but mainly located in M2 helices (Fig. 5*H*,*I*, only showing α1D-γ2E-β3A neighbor subunits for clarity). The latter may be due to the predicted projection of L302 towards the channel pore, which was absent in the open state (Fig. 5*G-I*, lower panels). It is noteworthy that γ2T310 is a residue that was perturbed independent of the conformational state of the receptor, whereas β3T281 was solely perturbed in the closed and desensitized states. These threonines correspond to the 6’ position in the M2 helix that outlines the channel pore. Highly conserved through all GABA_A_ subunits, T310 and T281 are located just below the gate of the channel (L9’ position) and shape the inner mouth of the channel pore.

### The pore mutation P302L destabilized the channel gate in the open state

**Figure 6.**
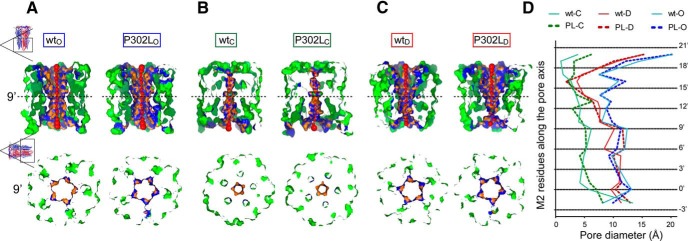
The pore mutation P302L destabilized the channel gate in the open state. ***A-C***, Visualization of pore sections in the open (***A***), closed (***B***), and desensitized (***C***) states were shown parallel to the membrane (top panels) and from the extracellular side (bottom panels) of wt and P302L structures. In the top panels, the section of each structure was obtained by cutting the protein structure along the pore-axis. Red spheres represented pore centers at given pore heights and their diameters correspond to 1/10 of the pore diameter calculated at that point. Bottom panels represent transverse sections of the pore-axis at the 9’ position (dashed lines in top panels), where the channel gate was located. Pore-lining side chains and residues at 3-Å steps within the cavity were colored in orange and blue, respectively. The remaining part of the transmembrane domain was shown in green. ***D***, Pore diameter profiles at 3-Å steps corresponding to the pore-lining residues in the open (***A***), closed (***B***), and desensitized (***C***) states of wt and P302L structures. Horizontal black dotted lines show positions of pore-lining residues in M2 (see Figure 1*A*). PL, P302L. Letters in subscript refer to open (O), closed (C), or desensitized (D) states.

To further investigate whether the γ2(P302L) subunit mutation altered the ion channel pore, we determined the structural characteristics of the channel cavity and the pore-lining residues along the axis of the channel pore through the implementation of two computational fully-automatic methods (Pellegrini-Calace et al., 2009; Masood et al., 2015). Six views of the transmembrane domain (M1 to M4) of the GABA_A_ receptor structure (Fig. 6*A-C*, top panels), which represent approximately 32 Å along the pore axis (i.e., *xz*-plane section), show the variation of pore diameters at 3-Å steps among wt and mutant structures in the open, closed, and desensitized states (Fig. 6*D*). In these pore visualization views, the top of the pore axis corresponds to the outermost part of the pore (∼20’ position), which is the extracellular vestibule of an asymmetric pore divided by the narrow open gate (9’ position). On the other hand, the bottom of the pore axis corresponds to the innermost part of the pore (-2’ position), which is the intracellular vestibule of the pore. As previously reported for GlyR (Du et al., 2015) and GluCL channels (Hibbs and Gouaux, 2011; Althoff et al., 2014), these observations support the concept that the shape of the pore of the GABA_A_ receptor also shows an asymmetrical hour-glass-like cavity. Considering the centers of the pore at 3-Å steps along the pore axis (Fig. 6*A-C*, red spheres), and their sizes being proportional to the pore diameter measured at that point, the pore diameter profile shows a great variability of both outer and inner vestibules among the structures (Fig. 6*D*). Taking into account the presence of these two vestibules, we compared the diameters of the cavity between the outermost position (21'-18' position) and the open gate (9' position), which represent the outer vestibule, and the latter and the innermost position (-2') that represents the inner vestibule, between wt and mutant structures for each conformational state. Figure 6*D* shows the differences of the pore diameter profiles along the pore axis of both outer and inner vestibules. Comparisons made against the wt structure (solid lines) revealed that although in the open state the mutated structure narrowed the outer vestibule, but increased the inner vestibule, in the closed state, the mutated structure narrowed the inner vestibule whereas increased the outer vestibule (dashed lines). Conversely, in the desensitized state, the mutated structure solely increased the inner vestibule. Moreover, cross-sectional views of the pore at the open gate (9’ position) (Fig. 6*A-D*, bottom panels) showed that in both open and closed states the open gate was reduced, whereas in the desensitized state, it was increased.

**Figure 7.**
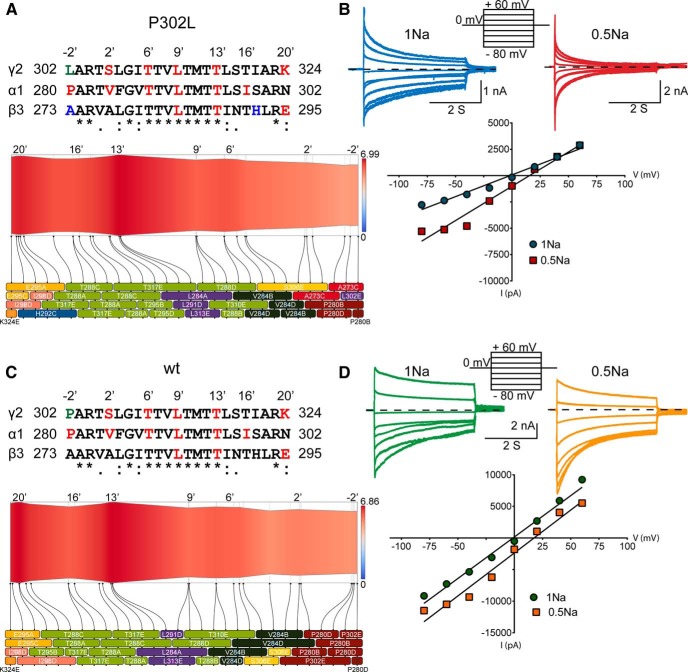
The pore mutation P302L had no effect on GABA_A_ receptor anion selectivity. ***A***, ***C***, Two-dimensional representations of channel radius (in Å) along the pore-axis for both P302L (***A***) and wt (***C***) structures in the open state, which was implemented by ChExVis. The analysis showed the pore-lining residues (colored boxes) numbered according to protein sequence and position in M2 of pentameric 3D GABA_A_ receptor structures with the subunit arrangement β3(chain A)-α1(chain B)-β3(chain C)-α1(chain D)-γ2(chain E). Above, alignments of the transmembrane M2 of GABA_A_ receptor γ2, α1, and β3 subunits showed the location and the conservation of the residues within M2 [identical (*), conservative (:), semiconservative (.)]. The residues were in concordance with the boxes below the 2D views. In red were common pore-lining residues that formed part of the inner face of the cavity predicted for both wt and P302L structures, while in blue were those only predicted for the P302L structure. In green, it was the site of the substitution. Below the 2D views, boxes were labeled and colored based on amino acid type as follows: E, glutamic acid (orange); T, threonine (light green); S, serine (yellow); A, alanine (red); I, isoleucine (rose); L, leucine (purple); V, valine (dark green); P, proline (brown); K, lysine (red orange); H, histidine (blue). The suffix after the residue number indicates the subunit to which the residue belongs. The dotted lines connecting the boxes represented the position of the residues along the pore-axis, as indicated on the top of the panels. ***B***, ***D***, Current-voltage (I/V) plots and corresponding GABA-gated peak current responses of mutant γ2L(P302L) (***C***) and wt (***D***) α1β3γ2L GABA_A_ receptors obtained from membrane potential range from -80 mV to +60 mV following rapid application of 1 mM GABA for 4s. The traces represented representative individual cells recorded in control (1 Na) or dilute (0.5 Na) solutions to determine the relative permeability of anions of mutant P302L and wt receptors.

These data suggested that in the open state, while the channel gate was constricted, the desensitized gate was enlarged. By analyzing the radius along the channel pore (Fig. 7*A*,*C*), the pore radius at the 9’ position was reduced to 5.18 Å in the P302L model in comparison with the wt structure model (5.28 Å). These subtle differences were accompanied by the rearrangement of two residues, not present in the wt structure, as outlining the channel pore. Both residues of the β3 subunit, H292 (17’ position) and A273 (-2’ position), were located at the outer and inner entrances of the pore, respectively. Although GABA_A_ receptors were formed by arrangement of α, β, and γ subunits, small differences in the entrances of the pore caused by a single substitution in the γ2 subunit might account for differences in how the ions pass through the channel. These observations indeed support our hypothesis that the occurrence of mutations at the intracellular entrance of the pore is relevant to the structural mechanisms that govern the gating of GABA_A_ receptors, as discussed in the next sections.

### The pore mutation P302L at the -2’ position did not affect GABA_A_ receptor anion selectivity

It is assumed that the movement of ions through the channel pore obeys the physical-chemical transport properties into barriers and wells (Bormann et al., 1987). Similar to other channels, GABA_A_ receptors have wide extracellular and intracellular entrances that lead to the channel gate. Thus, amino acid substitutions at these entrances, such as that caused by P302L, may cause a loss of the selectivity of ions entering the channel as discussed above. To gain insights into whether the predicted perturbations at the entrances of the channel pore could result in loss of selectivity to anions, GABA-gated peak current responses of α1β3γ2L(P302L) and wt α1β3γ2L GABA_A_ receptors at membrane potentials from -80 to +60 mV were measured in physiological (1Na) and diluted (0.5Na) extracellular solutions (Fig. 7*B*,*D*). In our experimental conditions, at the extracellular NaCl concentration of 142 mM (1Na), the theoretical V_rev_ for chloride given by the Goldman-Hodgkin-Katz equation is approximately -1.63 mV. The dilution of NaCl to 72 mM (0.5Na) predicts a relative right shift of the zero chloride current to V_rev_ approximately +13.04. Figure 7 shows representative I/V plots recorded from four different cells expressing receptors containing γ2L(P302L) subunits (Fig. 7*B*) and wt (Fig. 7*D*) receptors in physiological (P302L, 0.62 ± 1.07 mV, *n* = 3; wt, 0.02 ± 1.26 mV, *n* = 5; *p* = 0.7560, unpaired two-tailed Student’s *t* test) and diluted NaCl concentrations. Noticeably, I/V plots for P302L mutant and wt receptor currents showed a significant rightward shift in V_rev_ (*p* < 0.0001, one-way ANOVA with Dunnett’s and Tukey’s multiple comparisons test) with decreasing extracellular NaCl (P302L, 14.73 ± 0.52 mV, *n* = 7; wt, 13.99 ± 1.12 mV, *n* = 5; *p* = 0.5222), which demonstrated that both currents were carried by chloride ions and remained anion selective.

### Single-channel gating properties of GABA_A_ receptors were impaired by the γ2(P302L) subunit mutation

Our studies showed that depending on the state of the channels, whether they were in the closed or open states, the occurrence of the γ2(P302L) subunit mutation in the channel pore increased or decreased, in an asymmetrical manner, both outer and inner vestibules. Conversely, in the desensitized state, the mutation produced only an increase of the diameter of the inner vestibule of the pore, which widened the cytoplasmic side of the pore. In the open state, a decrease in the outer vestibule of the pore can affect the anticlockwise rotation of the transmembrane M2 segments for channel activation (Hibbs and Gouaux, 2011; Althoff et al., 2014; Du et al., 2015). In this regard, our data showed that the β3(H292) residue was among the pore-residues outlining the inner face of the cavity in the P302L model (Fig. 7*A*). It seems that H292, mapped at the γ2+/β3-interface in the wt model, is accessible to the pore in the P302L model, which might predicted by a clockwise tilt towards the channel pore, perturbing the anticlockwise rotation during channel activation. In addition, the narrowing of the inner vestibule of the pore in the closed state suggested that the channel was transiently trapped in a nonconducting state (Gielen et al., 2015). On the other hand, a failure of pore closure of the inner entrance in the desensitized state could lead to a partially nonconducting desensitized state, thus favoring occurrence of subconductance openings (Dani, 1986; Bormann et al., 1987; Root and MacKinnon, 1994). To determine whether the structural changes were correlated with the macroscopic kinetic defects and resulted in changes in the gating of the receptor, we measured the properties of GABA-evoked (1 mM) single-channel currents recorded from HEK293T cells coexpressing α1 and β3 subunits with wt γ2L or mutant γ2L(P302L) subunits.

**Figure 8.**
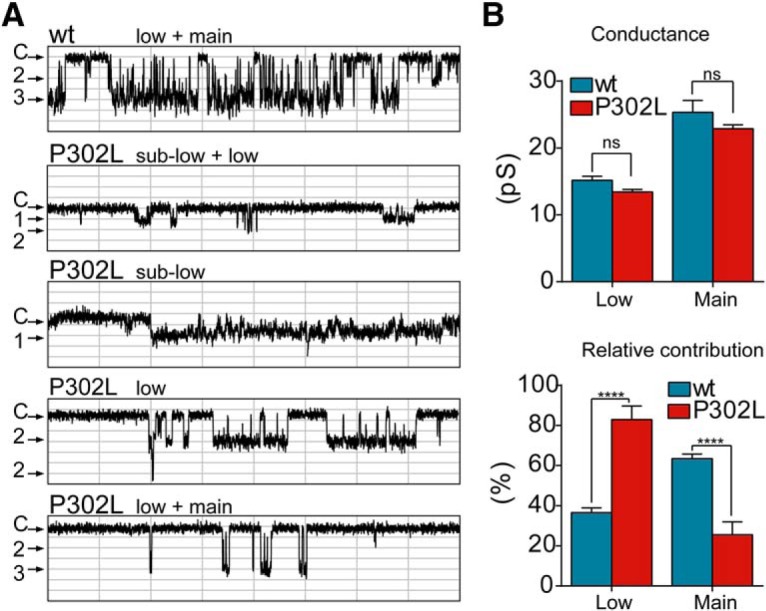
The *de novo* γ2(P302L) subunit mutation shifted the openings of GABA_A_ receptors to low-conductance states. ***A***, Representative single-channel current traces from cell-attached patches of wt α1β3γ2L and mutant α1β3γ2L(P302L) GABA_A_ receptors recorded from HEK293T cells. Patches were voltage clamped at +80 mV and continuously exposed to 1 mm GABA. Openings were downward and each representative trace was a continuous 1600-ms recording. Divisions in the *x*-axis corresponded to 200 ms, and in the *y*-axis to 0.5 pA. Note that the cluster of openings fell into the three distinct types of conductance: main (3), low (2), and sublow (1) openings. C refers to the closed state. ***B***, Means of the main and low conductance (pS) and the relative contribution (%) of each conductance level were calculated from patches of wt α1β3γ2L and mutant α1β3γ2L(P302L) GABA_A_ receptors displaying both levels of openings. Values were expressed as mean ± SEM. Two-way ANOVA with Sidak’s multiple comparisons test was used to determine significance. ****p < 0.0001, and ns, not significant (*p* = 0.7287), respectively. See Table 2 for details.

**Figure 9.**
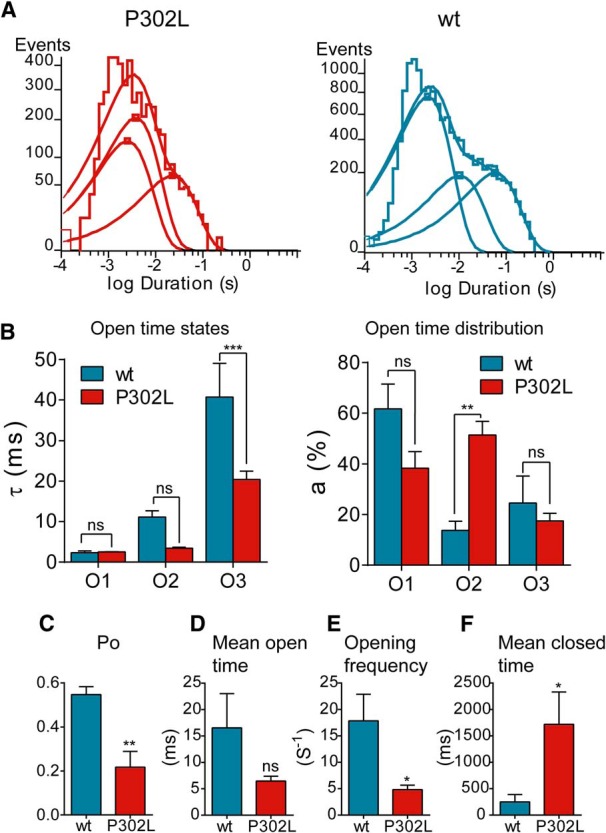
The *de novo* γ2(P302L) subunit mutation reduced GABA_A_ receptor single-channel currents. ***A***, Open time histograms for α1β3γ2L(P302L) and wt receptors were shown. ***B***, Summary bar graphs of time constants (τo1, τo2, and τo3) and representative areas (ao1, ao2, and ao3) of open time histograms in ***A*** for wt and mutant P302L receptors were shown. Values reported were mean ± SEM. Two-way ANOVA with Sidak’s multiple comparisons test relative to wt. ***p < 0.001, **p < 0.01, and ^ns^*p* > 0.05, respectively. ***C-F***, Bar graphs summarize the effects of α1β3γ2L(P302L) on open probability (Po) (***C***), mean open time (***D***), opening frequency (***E***), and mean closed time (***F***) of the receptor. Values were expressed as mean ± SEM. Statistical differences were determined using unpaired *t* test relative to wt. **p < 0.01, *p < 0.05, and ^ns^*p* > 0.05, respectively. See Table 2 for details.

Single channels from coexpressed α1β3γ2L subunits opened into brief bursts and frequent prolonged (>500 ms) burst clusters (Fig. 8*A*) and opened to a high main-conductance level of ∼21-28 pS and to a low-conductance level of ∼14-18 pS in all seven patches (Table 2), as described previously (Twyman et al., 1990; Fisher and Macdonald, 1997). In contrast, single-channel burst clusters from coexpressed α1β3γ2L(P302L) subunits occurred at three distinct conductance levels in 14 patches. Combination of clusters of main-conductance (∼22-25 pS) and low-conductance (∼13-15 pS) openings were observed in seven of the patches (Fig. 8*A*, Table 2). Low-conductance openings were reported in patches with αβ receptors (Lo et al., 2010). In addition, a novel sublow-conductance cluster type of openings of ∼7 pS (6.61 ± 0.15) occurred in combination with clusters of openings of ∼13-15 pS in seven of the α1β3γ2L(P302L) patches recorded (Fig. 8*A*). It was important to note that this type of sublow-conductance openings did not occur in wt receptors (Bormann et al., 1987; Twyman et al., 1990; Fisher and Macdonald, 1997). With the intent to simplify the kinetic analysis, those patches with sublow-conductance openings were excluded from further single-channel measurements. Therefore, we measured the single-channel properties of seven of the patches from cells coexpressing wt α1β3γ2L or mutant α1β3γ2L(P302L) receptors that exhibited only main- and low-conductance openings. When the openings were compared between wt and α1β3γ2L(P302L) receptors, no significant differences in conductance levels were observed (Fig. 8*B*, top panel; Table 2). Conversely, the relative distribution of main- and low-conductance openings that contributed to the behavior of α1β3γ2L(P302L) receptors was significantly different from the wt receptor. The γ2L(P302L) subunit mutation caused a significant shift in the contribution of these levels, increasing the contribution of the low-conductance openings (Fig. 8*B*, lower panel), while reducing the contribution of the main-conductance openings (Table 2). Both mutant and wt receptors opened to at least three different types of openings (O1, O2, and O3) with three open time constants (τ_o1_, τ_o2_, and τ_o3_) (Table 2), and open time distributions that were fitted best by three weighted (a_o1_, a_o2_, and a_o3_) exponential functions (Fig. 9*A*,*B*). Receptors containing the γ2L(P302L) subunit mutation had reduced open time constants (*p* = 0.022) and increased relative occurrence of the shortest open states (*p* = 0.004), with a slight reduction of single-channel mean open time (Table 2). In addition, mutant receptors reduced channel open probability (*p* = 0.002) and single-channel opening frequency (*p* = 0.039) and increased single-channel mean closed time intraburst (*p* = 0.029) compared with wt receptors (Fig. 9*C-F*, Table 2).

**Table 2.** Effects of γ2(P302L) subunit mutation on α1β3γ2L receptor single-channel properties

	***α1β3γ2L***	***α1β3γ2L(P302L)***	
**Single-channel properties**			***p*^a^**
Open probability	0.547 ± 0.036	0.217 ± 0.072	0.0015
Mean open time, ms	16.5 ± 6.49	6.46 ± 0.93	0.1500
Opening Frequency, S^−1^	17.8 ± 0.05	4.81 ± 0.86	0.0386
Mean closed time, ms	249 ± 141	1718 ± 613	0.0287
**Open time constants**			***p*^b^**
τ_O1_, ms	2.33 ± 0.46	2.53 ± 0.06	0.0215
τ_O2_, ms	11.2 ± 1.55	3.42 ± 0.29	
τ_O3_, ms	40.7 ± 8.34	20.4 ± 2.04
a_O1_, %	62 ± 10	38 ± 7	0.0040
a_O2_, %	14 ± 4	52 ± 5	
a_O3_, %	25 ± 11	18 ± 3
**Conductance**			***p*^b^**
Low openings, pS	15.16 ± 0.59	13.41 ± 0.38	0.7287
Main openings, pS	25.34 ± 1.77	22.89 ± 0.575
Low openings, %	36.57 ± 2.381	82.98 ± 6.731	0.0001
Main openings, %	63.43 ± 2.381	25.53 ± 6.392

Kinetic parameters of single-channel currents in cell-attached patches held at +80 mM with 1 mM GABA in the glass electrodes were obtained. The τ_s_ and a_s_ refer to the time constants and fractions of the three exponential components (O1, O2, and O3), which best represent the distributions of the single-channel openings. Values refer to combined data of low- and main-conductance openings reported are mean ± SEM (*n* = 7). ^a^Unpaired two-tailed Student’s *t* test relative to α1β3γ2L. ^b^Two-way ANOVA with Sidak’s multiple comparisons test relative to α1β3γ2L.

### The low-conductance γ2(P302L)-GABA_A_ receptors are zinc insensitive

As described above, the presence of low-conductance cluster type of openings suggested the formation of α1β3 receptors in the membrane of wt and α1β3γ2L(P302L) patches and support the notion that the observed difference in the gating of α1β3γ2L(P302L) receptors was the result of the kinetic behavior of a mixture of two types of channels. Thus, we computed the kinetic properties of each component separately to gain insight into the contribution of both main- and low-conductance openings in the gating of wt and mutant αβγ receptors and then compared with those that express only αβ receptors. As shown in Table 3, it was obvious that wt and mutant αβγ receptors were not composed of αβ receptors due the presence of prolonged type 3 openings (O3) in both main- and low-conductance openings, which is characteristic of receptors assembled from αβγ subunits. Conversely, the lack of O3 openings together with the presence of brief O1 type of openings are distinct properties of αβ receptors, which makes the single-channel behavior of these receptors faster than αβγ receptors. These comparisons clearly showed that the type of receptor containing the γ2L(P302L) subunit mutation had kinetic properties that differed from receptors assembled with αβ subunits alone. When the two types of conductance openings between wt and mutant receptors were compared, it was notable that low-conductance openings were contributing largely to the kinetic behavior displayed by mutant receptors. Notwithstanding the presence of the three types of openings in the low-conductance state, these openings were briefer than in the wt receptor, which decreased by 70% the mean open time in the mutant receptor. Unexpectedly, main-conductance types of openings were more prolonged in the γ2L(P302L) mutant receptors than wt receptors, but this was not deemed significant, because of the small contribution (∼13%) of total channel openings. We can conclude that α1β3γ2L(P302L) receptors open mainly through clusters of bursts of low-conductance openings.

**Table 3.** Distribution of main and low channel openings of GABA_A_ receptor

	***α1β3γ2L***		***α1β3γ2L(P302L)***		***αβ*^a^**
	Main	Low	Main	Low	Low
Number of openings	9938	7086	606	3929	9723
Open probability	0.555	0.619	0.077	0.149	0.680
Mean open time, ms	9.89	14.7	16.8	5.01	2.43
Opening components, ms					
τ_O1_ (%)	2.71 (80)	1.67 (57)	4.06 (33)	2.65 (62)	1.06 (93)
τ_O2_ (%)	14.3 (9)	8.13 (23)	20.7 (63)	4.99 (31)	6.39 (7)
τ_O3_ (%)	55.3 (11)	55.8 (20)	54.5 (4)	23.9 (7)	*NO*

^a^Single-channel properties of αβ receptors recorded from cell-attached patches held at +80 mM with 1 mM GABA are shown for comparison. *NO*, not observed.

**Figure 10.**
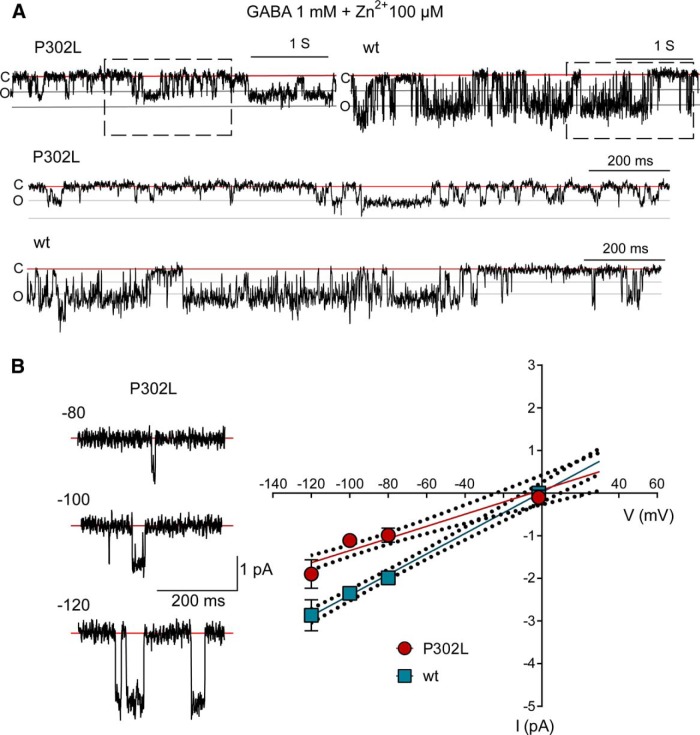
The low-conductance γ2(P302L)GABA_A_ receptors were zinc insensitive. ***A***, Single-channel current traces from cell-attached patches of mutant γ2L(P302L) and wt recorded at +80 mV and continuously exposed to 1 mM GABA and 100 µM zinc. Cluster openings were downward. Upper panels were representative traces continuously recorded for 4000 ms. Red lines indicated that the channel was closed (C), while the gray lines indicated when the channel was open (O) in steps of 1 pA. Dashed boxes enclosed an expanded section of 1600 ms, which was shown below as indicated. ***B***, Representative low-conductance current traces from cell-attached patches of mutant γ2L(P302L) receptors recorded at various membrane potentials in the presence of 1 mM GABA and 100 µM zinc. For the linear regression analysis of conductance, the membrane potentials were indicated as negative values. The traces were recorded from the same patch at the membrane potentials indicated. The red lines represented the closed state of the channels. In the right panel, single-channel current-voltage (I/V) plots of mutant γ2L(P302L) and wt receptors were shown. The amplitude of the most frequently occurring opening was used for the construction of the I/V plots. Thus, low-conductance openings were used for the mutant receptor, whereas main-conductance openings were used for wt receptors. Data points were values expressed as mean ± SEM (*n* = 3-5 cells for each experimental condition). Solid lines represented fits of the linear regression equation, and the doted lines represented 95% confidence intervals of the best-fit line. Zn^2+^, zinc.

Further, to confirm that the low-conductance openings resulted from tertiary α1β3γ2L(P302L) receptors, we analyzed the properties of GABA-evoked single-channel openings measured in the presence of 100 µM zinc (Fig. 10). Zinc is a well-known GABA_A_ receptor antagonist whose potency is affected by the subunit composition of the receptor (Smart et al., 1991; Hosie et al., 2003). Thus, receptors lacking γ subunits are strongly inhibited by zinc. Whether low-conductance openings occurred due to the presence of binary αβ receptors, it is expected to be block by zinc. Noteworthy, we found that, in the presence of zinc and at a membrane potential of +80 mV, the activation of α1β3γ2(P302L) receptors occurred mainly as clusters of low-conductance openings (12.30 ± 0.92 pS, *n* = 5, *p* = 0.240 unpaired *t* test) (Fig. 10*A*,*B*), which was comparable with the results obtained previously (Table 2). In addition, no differences were found for wt α1β3γ2 receptors (24.84 ± 0.46 pS, *n* = 3, *p* = 0.863 unpaired *t* test).

Up to this point, we have shown that most of mutant channels open with a lower conductance than wt channels, and that this was not due either to alterations in channel permeability for chloride ions or the presence of binary receptors on the cell surface. These findings also suggested that the conductance of the mutant channel is expected to be a linear assuming that the chloride V_rev_ was ∼0 mV. Figure 10*B* shows GABA-evoked single-channel openings at various membrane potentials in the presence of 100 µM zinc. At membrane potentials of +100 mV and +120 mV, the γ2L(P302L) mutant receptors displayed openings with a conductance of 13.38 ± 0.35 pS (*n* = 5, *p* < 0.0001 unpaired *t* test) and 23.72 ± 1.58 pS (*n* = 5, *p* = 0.004 unpaired *t* test), which were distinguishable from the wt receptor (29.38 ± 0.97 pS, *n* = 3; and 35.83 ± 2.63 pS, *n* = 3, respectively). As a result, low-conductance openings for mutant receptors had a slope conductance of 14.24 ± 1.72 pS, which was significantly different from wt receptors (24.17 ± 1.18 pS, *p* = 0.000165).

In line with the structural modeling, these results demonstrated that mutant α1β3γ2L(P302L) receptors might perturb the conduction pathway and destabilize the open conformation while trapping the receptor in nonconducting closed and desensitized conformations. This is in accord with reductions in maximum currents, the increased relative contribution of low (and sublow) conductance openings over the main-conductance openings, the enhanced desensitization, slowed activation, and accelerated deactivation in functional experiments.

### GABA_A_ receptor-mediated mIPSCs were reduced by overexpressing γ2(P302L) subunits in cultured neurons

**Figure 11.**
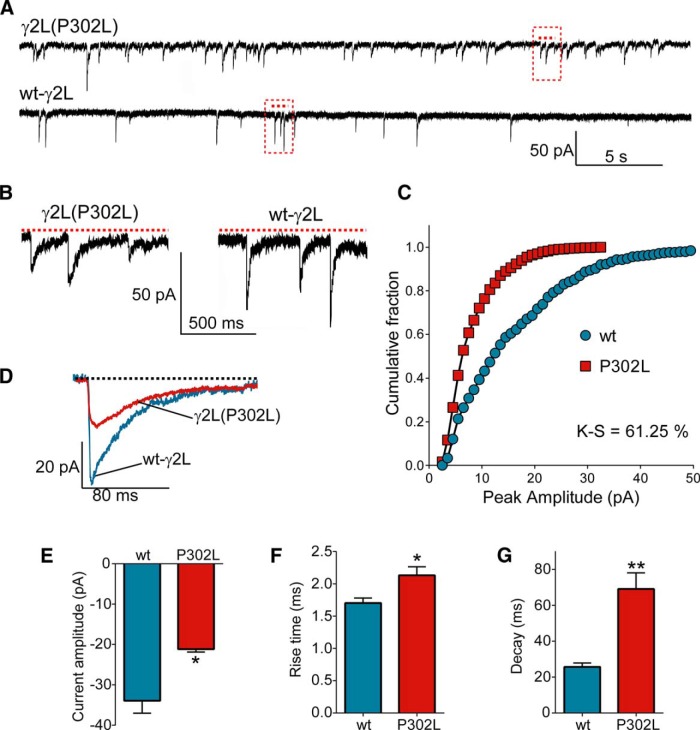
GABA_A_ receptor-mediated mISPCs were altered by overexpression of γ2(P302L) subunits in cultured neurons. ***A***, Representative GABA_A_ receptor-mediated mISPCs traces recorded at -60 mV in cultured cortical neurons overexpressing mutant γ2L(P302L) subunits or wt γ2L subunits. ***B***, A segment of each raw trace in A (red dashed lines) was expanded for better visualization of the differences in mISPC properties. ***C***, Cumulative histograms of GABA_A_ receptor-mediated mISPCs current amplitudes of neurons overexpressing mutant γ2L(P302L) subunits or wt γ2L subunits show the Kolmogorov--Smirnov (K-S) comparison test with a statistical value of 61% (*p* < 0.00001). ***D***, Overlapped ensemble average traces of mISPCs recorded from receptors containing wt-γ2L (blue) and mutant γ2L(P302L) (red) subunits showed reduced amplitude and kinetic changes. ***E***, ***F***, Bar graphs summarize the effects of overexpression of wt and mutant γ2L(P302L) subunits on current amplitude (***E***), rise time (***F***), and decay (***G***) of GABA_A_ receptor-mediated mISPCs. Values were expressed as mean ± SEM. Statistical differences were determined using unpaired *t* test relative to wt. **p < 0.01 and *p < 0.05, respectively.

Our data demonstrated that the γ2(P302L) subunit mutation significantly decreased the function of GABA_A_ receptors expressed in HEK293T cells, also suggesting that there must be an impairment of their function at GABAergic synapse. As previously described (Swanwick et al., 2006), widespread expression of clusters of GABA_A_ receptors containing αβγ subunits at GABAergic synapses was reached in mature cultured neurons around DIV 19-21. To determine whether the mutant γ2(P302L) subunit altered the function of GABAergic synapses, we overexpressed wt γ2L or mutant γ2L(P302L) subunits in cultured cortical neurons at DIV 12 and measured current amplitude and kinetic properties of GABA_A_ receptor-mediated mIPSCs at DIV 19-21 (Fig. 11). Thus, GABA_A_ receptor-mediated mIPSCs recorded after overexpression of wt γ2L subunits displayed current amplitude (-33.94 ± 3.08 pA, *n* = 4), rise time (1.70 ± 0.08 ms), and decay (25.63 ± 2.23 ms) values that suggested the transfected wt γ2L subunits formed functional receptors with endogenous αβ subunits, resembling mIPSCs yielded at the GABAergic synapse (Fig. 11). In contrast, overexpression of mutant γ2L(P302L) subunits decreased current amplitude (-21.16 ± 0.72 pA, *n* = 4, *p* = 0.0156) and slowed rise time (2.13 ± 0.14 ms, *p* = 0.049) and decay (69.11 ± 8.96 ms, *p* = 0.009). These results suggest that mutant γ2L(P302L) subunits were incorporated into GABAergic synapses and caused a dominant negative effect, reducing GABAergic function. Although the type of receptor expressed in the membrane was unknown since multiple GABA_A_ receptor subunits were expected to be coexpressed (Swanwick et al., 2006), these findings were comparable with the gating deficiencies found in α1β3γ2L(P302L) receptors coexpressed in HEK293T cells.

## Discussion

The findings presented here shed light onto a novel mechanism in the pathogenesis of Dravet syndrome. This study identified a missense *de novo* mutation in the GABA_A_ receptor γ2 subunit, P302L, in a patient with Dravet syndrome. The first question to consider was whether a single missense mutation was associated with this catastrophic epileptic encephalopathy. It is well known that missense mutations in coding sequences in the GABA_A_ receptor γ2 subunit gene *GABRG2* are associated with relatively mild epilepsy phenotypes, including childhood absence epilepsy and febrile seizures (Wallace et al., 2001; Audenaert et al., 2006; Shi et al., 2010), and with the genetic epilepsy with the febrile seizures plus (GEFS+) spectrum (Baulac et al., 2001). Intriguingly, missense mutations in the GABA_A_ receptor α1 subunit gene have been reported in cases of Dravet syndrome (Carvill et al., 2014). Taking into account the location of these mutations in the α1 subunit, it is significant that all three mutations (R112Q, G251S, and K306T) share the same topological domain in the receptor, which is directly related to the ligand-binding coupling mechanism (Bianchi et al., 2001; Bera et al., 2002; Keramidas et al., 2006; Wang et al., 2010; Calimet et al., 2013; Althoff et al., 2014). Thus, α1(R112Q) and α1(G251S) subunit mutations face the β+/α- subunit-subunit GABA-binding interface within the N terminus and M1 transmembrane helix, respectively, whereas the α1(K306T) subunit mutation is located in the M2-M3 extracellular loop, which is part of the coupling interface of the GABA_A_ receptor. In line with these findings, the next question to consider was whether the location of the γ2 subunit mutation in the GABA_A_ receptor may account for the severity of the epilepsy phenotype. While most of the missense γ2 subunit mutations are preferentially distributed across the N-terminal domain and in the outermost region of the transmembrane segment M2 not facing directly β+/α- GABA-binding coupling interface (Baulac et al., 2001; Wallace et al., 2001; Audenaert et al., 2006; Shi et al., 2010; Lachance-Touchette et al., 2011; Carvill et al., 2013; Reinthaler et al., 2015), the γ2(P302L) subunit mutation was mapped in the pore-forming region of the GABA_A_ receptor. This was probably the most significant feature that distinguished the defects caused by the occurrence of this mutation as discussed below.

### A mechanism of gating impairment of GABA_A_ receptors by the γ2(P302L) subunit mutation

For Cys-loop receptors (Hibbs and Gouaux, 2011; Althoff et al., 2014; Du et al., 2015), pore-lining residues of the transmembrane M2 domain of GABA_A_ receptors form the axis of the channel pore. The pore exposes two gates that are open or closed depending on the conformational state of the receptor. One gate corresponds to the activation/opening gate, which is at position 9’, and the other in the cytoplasmic interface representing the desensitization gate at position -2’ (Fig. 5). Thus, the conducting pathway shows an asymmetrical hour-glass-like cavity divided by the narrow open gate at 9’ position. The top of the pore axis corresponds to the outermost part of the pore, which is the extracellular vestibule, and the bottom of the pore axis (-2’ position) corresponds to the innermost part of the pore, which is the intracellular vestibule, where the γ2(P302L) subunit mutation occurs. Through structural simulation of the different states that the GABA_A_ receptor adopts during the activation and deactivation of the channel, we found that the shapes of the vestibules that determine the features of the pore were deeply affected by the mutation, thereby altering the gating of the receptor. Further, we propose a structural mechanism to explain the deficiencies observed in the gating of receptors containing the γ2(P302L) subunit mutation. Thus, the decrease in the outer vestibule of the pore in the open state can affect the anticlockwise rotation of the transmembrane segments M2 for channel activation (Hibbs and Gouaux, 2011; Althoff et al., 2014; Du et al., 2015) and may account for the slowing in the activation of the channel and the occurrence of low-conductance openings. The narrowing of the inner vestibule of the pore in the closed state instead suggests that the channel is transiently trapped in a nonconducting state (Gielen et al., 2015), explaining the profound desensitization of the currents. Finally, the pore closure failure at the inner entrance in the desensitized state could lead to a partially nonconducting desensitized state, favoring the occurrence of sublow-conductance openings (Dani, 1986; Bormann et al., 1987; Root and MacKinnon, 1994), corroborating the experimental observations of clusters of sublow openings. Further, slowing of recovery from desensitization and reduction in the activation caused by the γ2(P302L) subunit mutation may be responsible for facilitating the allosteric modulation by zinc (Barberis et al., 2000) and increase of zinc sensitivity.

### Missense epilepsy mutations in the M2 domain of Cys-loop receptors cause hyperexcitability by altering current desensitization and channel conductance

Among Cys-loop family receptors, structural studies revealed that the nicotinic acetylcholine receptor (nAChR) (Unwin, 2005), GABA_A_ receptor (Miller and Aricescu, 2014), GlyR (Du et al., 2015), and GluClR (Hibbs and Gouaux, 2011) share a common ion-conducting pore scaffold composed of four transmembrane segments (M1 to M4), with the transmembrane segment M2 lining the pore. Thus, it has been suggested that these receptors share a similar structural basis for the ligand binding-channel gating coupling mechanism (Unwin, 2005; Hibbs and Gouaux, 2011; Althoff et al., 2014; Miller and Aricescu, 2014; Du et al., 2015). Missense mutations of the nAChR α4 subunit associated with autosomal dominant nocturnal frontal lobe epilepsy affected highly conserved residues in M2, specifically those residues predicted to be part of the M2-axis that rotates when the agonist binds and opens the pore. Similar to the defects caused by the GABA_A_ receptor γ2(P302L) subunit mutation, nAChR α4(S248F) (Weiland et al., 1996; Kuryatov et al., 1997) and α4(S252L) (Matsushima et al., 2002) subunit mutations caused faster desensitization and decreased single-channel conductance. In addition, in homomeric GlyR α1 subunits, autosomal dominant mutations associated with hyperekplexia were also found clustered in and around the pore-lining transmembrane segment M2 impairing GlyR α1 subunit gating. At the extracellular end of the M2, GlyR α1(R271Q) and GlyR α1(R271L) subunit mutations reduced single-channel conductance (Langosch et al., 1994), while the GlyR α1(V280M) subunit mutation in the M2-M3 loop enhanced glycine sensitivity and spontaneous channel activity (Bode et al., 2013). The GABA_A_ receptor γ2(P302L) subunit missense mutation occurred in the evolutionarily conserved proline residue in the pore region of GABA_A_ receptors, GlyRs (Du et al., 2015) and GluCL receptors (Hibbs and Gouaux, 2011). Remarkably, the autosomal dominant hyperekplexia GlyR α1(P250T) subunit mutation occurred at a position homologous to that of the γ2(P302L) subunit mutation. Resembling the γ2(P302L) subunit mutation, the GlyR α1(P250T) subunit mutation reduced glycine-activated current amplitudes, induced fast desensitization and reduced single-channel conductance (Saul et al., 1999), which accounted for the hyperekplexia phenotype. None of these mutations impaired cell surface expression. Thus, the main mechanisms by which these mutations cause hyperexcitability appear to be through impairment of receptor gating, thereby contributing to the epilepsy phenotype.

### Epileptogenic role of GABA_A_ receptor desensitization in shaping fast inhibitory synaptic currents

At inhibitory synapses, GABA_A_ receptors containing γ2 subunits are mainly synaptic receptors that mediate fast inhibitory postsynaptic currents (Farrant and Nusser, 2005). The γ2 subunit is required for postsynaptic GABA_A_ receptor clustering (Essrich et al., 1998), and postsynaptic GABA_A_ receptors control the time course of IPSCs, which represents the major mechanism of inhibition in the brain. GABA_A_ receptor desensitization has been postulated as a control mechanism in cases of repetitive firing (Jones and Westbrook, 1996), whereby subsequent stimulation is inhibited in order to maintain the balance between excitation and inhibition. Thus, the entry of the receptor into a desensitized conformation trapped the receptor bound to the agonist although no current flows through the channel, which favored late channel reopenings and prolonged the time course of IPSCs (Jones and Westbrook, 1995; Bianchi et al., 2001; Bianchi et al., 2007). We demonstrated *in vitro* in transfected HEK293T cells that the mutated γ2L(P302L) subunit assembled with other subunits and was effectively trafficked to the cell membrane and that the expressed mutant subunit enhanced macroscopic desensitization, reduced channel open probability and reduced frequency of GABA_A_ receptor single-channel openings. In cortical neurons, the mutant the γ2L(P302L) subunit was assembled with endogenous α and β subunits on the surface of the membrane and reduced currents and slowed decay of GABA_A_ receptor-mediated mIPSCs. Thus, the incorporation of the mutant subunit in the GABAergic synapse generated slow-rising and slow-decaying synaptic currents as a negative feedback mechanism due to the substantial desensitization of the receptor. Consequently, the GABAergic inhibition response for a given excitatory input would be limited, which might increase neuronal excitability. Mice with heterozygous deletion of *Scn1a* that reduced sodium currents in GABAergic inhibitory interneurons recapitulated many of the clinical features observed in patients with Dravet syndrome (Yu et al., 2006; Tai et al., 2014), suggesting that the functional impairment of inhibitory interneurons were likely to cause the epilepsy phenotype.

Overall, this study is a step forward for understanding the pathogenesis of Dravet syndrome and provides an additional physio-pathological mechanism that had not been considered before.

## Synthesis

Reviewing Editor: Jun Chen, University of Pittsburgh Decisions are customarily a result of the Reviewing Editor and the peer reviewers coming together and discussing their recommendations until a consensus is reached. When revisions are invited, a fact-based synthesis statement explaining their decision and outlining what is needed to prepare a revision will be listed below. The following reviewer(s) agreed to reveal their identity: Yongchang Chang

Your study provides some new mechanistic information on the cause of Dravet syndrome. Both reviewers indicated that your study is thorough, results are convincing, and the manuscript is well written. However, each reviewer offered some suggestions on how to strengthen your conclusions. While most of their comments should be straightforward to address, a few additional minor experiments may be needed. I copy the reviewers' comments at the bottom of this message.

Reviewer #1:

This manuscript investigates the consequences of a single residue change observed in a patient with Dravet syndrome and links this to changes in channel conductance (though see below) and gating parameters. The overall impression is that this is a thorough and convincing study complemented by a well written manuscript.

However, please find below a number of questions that need to be addressed:

1. How prevalent is the γ2-P302L mutation? Has it so far only been found in one individual? Do you think it could be a common mutation in patients with Dravet? Do you believe that other Dravet mutations will all lead to similar defective changes in GABAaR function? A brief discussion would inform the reader how general or case-specific you believe these findings to be.

2. Figure 1B: Please show the affected proline to leucine codon more clearly. The sequencing readouts above the nucleotide spectra seems to be the same for all three individuals? And in the affected II-3, it looks like a blue (C) and red (T) line is overlaid. The panel needs to show the mutation more clearly.

3. Regarding the relatively slow solution exchange time in whole cell recordings in combination with the lack of information on whole-cell capacitance and series resistance compensation, are you recording the peak currents without underestimation? Particularly relevant for the 'reduced' amplitude for the fast-desensitising P302L current.

4. Fig. 2B: Are the decays for P302L multi-exponential? If so, do you see differential effects of the mutation on desensitization?

5. Lines 332 and 347: Desensitization gate location at -2' for GABA receptors should refer to Gielen et al 2015 Nature Comm paper.

6. Use 'modelled' rather than 'simulated'.

7. Is it possible that recombinant expression levels of GABAaR subunits in hek cells may not reflect assembly patterns in neurons. For example, is there a possibility of alpha,beta assembled receptors?

8. You see a higher proportion of low conductance levels with the P302L mutation than you do with wildtype. Please assess if this could be due to a higher proportion of alpha,beta receptors as mutated subunits (g2-P302L) are often less willing to assemble in channel pentamers. To do this I suggest that you try to block alpha,beta channel openings with a selective Zn2+ concentration. If this is possible, the openings you are left with should only come from fully assembled abg(P302L) receptors.

9. Modelling: The images G-J are hard to resolve by eye as is the pore lining analysis with dashed lines looking similar to solids, please improve clarity.

10. Line 334: Not sure this part of the text 'clarifies' the mechanism. The findings might 'suggest' a mechanism....

11. Line 342: We 'modelled.....'

12. Line 352: Which residues are affected in alpha1 and beta 3 subunits by propagation? Also it is hard to see the changes in figs D-F, images are too small. Is the ordinate in J in units of As?

13. Line 380: By 'lessened', do you mean the pore diameters are reduced? The effect of P302L reducing the inner vestibule (diameter) is relying on just one point - can you corroborate this as all the other points in this area (up to '0') for both WT and mutant are overlaid.

14. The modelling studies need to remind the reader of the caveat that you are using the GlyR as a template. If the desensitized state of the GABAR beta3 subunit was used instead as a template for desensitized P302L receptor, would you see similar constrictions at -2'?

15. Line 434: Mean closed time is this for closed times within bursts, presumably most patches had more than one channel present? How did you determine the channel conductance if Vm is unknown?

16. Line 458: Narrowing the conductance pathway may be too simplistic. Main state conductances are still observed so if there is narrowing it must be dynamic to allow main state conductances.

17. Title & Line 20 & elsewhere in text: An important point of interpretation. The conductance of the channel is unaltered for P302L if I understand correctly, but the relative levels of low to main states are altered. If so, the statement in line 20 and the title needs to be modified with regard to channel conductance.

18. Line 592: For the recordings of mPSCs for the mutant receptors, Fig 8G shows the decays are prolonged not curtailed, (correct?) - can you clarify/correct the text here?

19. Lines 528-532: What evidence is there that the mutation affects subunit rotation?

20. Line 536: Presumably from the single channel recordings, the channel must be capable of closing as the current returns to the baseline. If it is trapped in a partially closed state, it must transient?

Reviewer #2:

GABAA receptors are the major inhibitory neurotransmitter receptors for fast synaptic inhibition in the brain. Mutations of the GABAA receptor subunits, especially the gamma2 subunit, have been found to be associated with different types of epilepsies. The P302L mutation of the gamma2 subunit described in this manuscript in a Dravet syndrome patient represents a novel finding. It is interesting that the mutation in the selectivity filter can dramatically alter gating behavior and redistribute gating states. The experiments are well-designed and conducted. The results are convincing. Data are properly interpreted. The manuscript is well written. I have the following comments:

1. GABA concentration responses: A mutation that changes a channel gating behavior also likely alters the sensitivity of the receptor to its agonist, since the binding affinity of an agonist to its receptor is state dependent. Thus, it is necessary to perform an experiment for GABA concentration response of the mutant alpha-beta-gamma receptor and compared it to those for the wild type and alpha-beta GABAA receptor.

2. Selectivity: The presence or absence of the -2'M2 proline can influence ionic selectivity (Galzi, et al, 1992), potentially by altering the orientation of the charged residues. Since the cis-trans isomerization (or conformation) of proline can potentially result in a large conformational change in the channel-lining domain M2, it is possible that a mutation of this proline can alter ionic selectivity of the channel. Thus, it would be interesting to see whether P302L can influence the ionic selectivity. That is, whether the reversal potential can be altered in the mutant channel when 50% of a cation in the extracellular solution is replaced by a non-permeant cation.

3. The visual abstract may not accurately reflect the mechanism of the mutation influence on channel function. First, the single channel data have 3 open states, but the kinetic model has only one open state. Second, the structural simulation and cartoons do not match. According to Figure 5J, in the open state, the pore diameter of the mutant is the same as that for wild type at 20' (not shortened as in the visual abstract). In the closed state, Figure 5J also shows that the pore diameter of the mutant is the same as that for the wild type at 9' position. Although the authors speculate that incomplete closure of the pore could be the mechanism of sub-conductance openings, this interpretation cannot serve as a conclusion in the abstract. In the desensitized state, at -2' position, Fig5J shows that both WT and mutant have the same diameter, but the mutant receptor has smaller diameter at -1' position. Since the observed mutation-induced changes are mainly in channel gating kinetics, the structural modeling in each state with a static model may not accurately reflect the changes in gating behavior change. To simulate the related changes, authors may need to do molecular dynamics simulation. Based on above comments, it is better without the visual abstract.
